# Soluble Amyloid-beta Aggregates from Human Alzheimer’s Disease Brains

**DOI:** 10.1038/srep38187

**Published:** 2016-12-05

**Authors:** Thomas J. Esparza, Norelle C. Wildburger, Hao Jiang, Mihika Gangolli, Nigel J. Cairns, Randall J. Bateman, David L. Brody

**Affiliations:** 1 Department of Neurology, 660 South Euclid Avenue, Box 8111, Washington University, St. Louis, Missouri, USA; 2 Department of Biomedical Engineering, Washington University, St. Louis, Missouri, USA; 3 The Knight Alzheimer’s Disease Research Center, Washington University, St. Louis, Missouri, USA; 4 Hope Center for Neurological Disorders, Washington University, St. Louis, Missouri, USA

## Abstract

Soluble amyloid-beta (Aβ) aggregates likely contribute substantially to the dementia that characterizes Alzheimer’s disease. However, despite intensive study of *in vitro* preparations and animal models, little is known about the characteristics of soluble Aβ aggregates in the human Alzheimer’s disease brain. Here we present a new method for extracting soluble Aβ aggregates from human brains, separating them from insoluble aggregates and Aβ monomers using differential ultracentrifugation, and purifying them >6000 fold by dual antibody immunoprecipitation. The method resulted in <40% loss of starting material, no detectible *ex vivo* aggregation of monomeric Aβ, and no apparent *ex vivo* alterations in soluble aggregate sizes. By immunoelectron microscopy, soluble Aβ aggregates typically appear as clusters of 10–20 nanometer diameter ovoid structures with 2-3 amino-terminal Aβ antibody binding sites, distinct from previously characterized structures. This approach may facilitate investigation into the characteristics of native soluble Aβ aggregates, and deepen our understanding of Alzheimer’s dementia.

Clinically defined dementia of the Alzheimer’s type (DAT) is the most common cause of age-related progressive cognitive dysfunction. Post-mortem, pathologically defined Alzheimer’s disease (AD) is present in the majority of patients diagnosed during life with DAT. The ‘Amyloid Cascade Hypothesis’, a leading idea regarding the cause of DAT, is derived from genetic studies of both age-related and familial early onset disease, both of which implicate increased production and aggregation of the Aβ peptide^1^,^2^. Thus, Aβ has been the major target for disease-modifying therapeutic development. However, the extent of Aβ deposition correlates only modestly with dementia; many middle aged and elderly people have extensive plaque deposition without any signs of dementia^3^,^4^,^5^,^6^,^7^. Furthermore, Aβ deposition begins decades before the onset of dementia^8^.

In recent years, water-soluble Aβ aggregates (varyingly termed Aβ oligomers, amyloid derived diffusible ligands, Aβ*56, amylospheroids, annular protofibrils, and other appellations) have been implicated more directly in causing synaptic dysfunction and neuronal cell death *in vitro*^9^,^10^,^11^,^12^,^13^,^14^,^15^,^16^ as well as impaired behavioral performance in animal models^17^,^18^,^19^. Soluble Aβ aggregates have been detected in human brain lysates from AD patients^15^,^20^,^21^,^22^,^23^,^24^,^25^,^26^,^27^,^28^,^29^,^30^,^31^,^32^,^33^,^34^,^35^. However, these aggregates are highly heterogeneous^15^,^23^,^24^,^26^,^27^,^28^,^32^,^35^,^36^,^37^,^38^,^39^, and it is unclear whether the characteristics of the soluble Aβ aggregates in the human brain are similar to the soluble Aβ aggregates in animal models or *in vitro* preparations^37^. Of note, native human brain soluble Aβ aggregates are orders of magnitude more toxic than similar size synthetic aggregates^24^,^27^. Furthermore, determining the relationship between human patients and animal models is of vital importance because to date, Aβ-targeted therapeutics developed using these animal models have not been successful at reversing DAT or altering disease progression.

Our group reported development of a sensitive, specific, quantitative, and high-throughput assay for soluble Aβ aggregates^40^ (termed ‘oligomers’ in that publication). The assay uses the monoclonal antibody HJ3.4 which is specific for the canonical N-terminus of Aβ^40^; it does not recognize amyloid precursor protein, unlike other commonly used antibodies such as 6E10 or 4G8. Using this assay, we were able to fully distinguish between DAT patients and high pathology non-demented controls with no overlap between groups based on the ratio of soluble Aβ aggregates to plaque area^40^.

However, a major challenge has been that the specific forms of soluble Aβ aggregates most relevant to human disease have not been determined. Aβ can potentially aggregate into a vast number of forms, consisting of different numbers of Aβ peptides, various size forms of Aβ, multiple Aβ post-translational modifications and alternative structural configurations of Aβ. It has not been clear which of these aggregation forms are most relevant to AD. Here we report a method to purify soluble Aβ aggregates directly from frozen human AD brain tissue, reasoning this would be the most relevant source for the species directly underlying dementia in humans.

## Results

**Figure 1:**
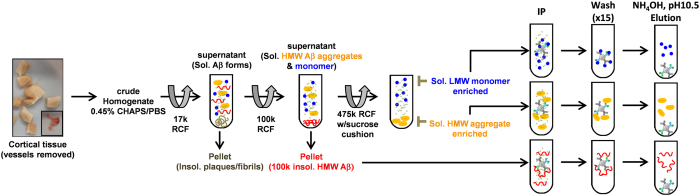
Method for isolating and purifying soluble Aβ aggregates from human AD brain. Cortical tissue was dounce homogenized in sub-critical micelle concentration of the detergent CHAPS, size forms of Aβ were isolated by differential ultracentrifugation, then Aβ was purified by dual antibody immunoprecipitation and elution in ammonium hydroxide. RCF: relative centrifugal force. Sol.: soluble, LMW: low molecular weight, HMW: high molecular weight. IP: immunoprecipitation.

Our approach to isolating soluble Aβ aggregates from human brain involved tissue homogenization, differential ultracentrifugation, and dual antibody immunoprecipitation (Fig. 1). During the methods development phase, we broke the problem down into three tasks: 1) Maximizing extraction of soluble Aβ aggregates from human Alzheimer’s disease brain tissue; 2) Isolating the soluble Aβ aggregates from other forms of Aβ; 3) Separating the soluble Aβ aggregates from other proteins.

We tested multiple different methods to address each of these tasks, and used four criteria to quantitatively assess the overall results: 1) Quantitative completeness of separation of the soluble Aβ aggregates from soluble monomers and insoluble aggregates; 2) Fold enrichment of Aβ compared to total protein; 3) Quantitative completeness of the recovery of the soluble Aβ aggregates present in the starting material, i.e. minimization of loss during purification; and 4) Minimization of *ex vivo* aggregation or disaggregation of Aβ during the extraction and purification process.

For quantification of soluble Aβ aggregates at each step of the process, we used a 96 well plate-based assay (Supplementary Fig. 1A) modified from our previously reported 384 well plate assay^40^. The soluble Aβ aggregate assay uses the same antibody, HJ3.4, to capture and detect Aβ. Monomeric Aβ is captured but not detected due to epitope masking by the capture antibody. For quantitation, the assay uses synthetic Aβ dimers, so the quantitative units are ‘dimer equivalents’ even though the assay measures soluble aggregates of many sizes. This assay has a lower limit of quantitative reliability of 31.25 pg/mL dimer equivalents and an approximately 100-fold dynamic range. It is relatively high throughput, such that a single investigator can run up to 6 plates per day. However it only recognizes aggregates with ≥2 free canonical N-termini and thus should be considered an assay for just this class of soluble Aβ aggregates. At each step of the process, we also quantified total Aβ with a free N-terminus (Aβ_1−x_) using a different 96 well plate-based ELISA with HJ5.1, a mid-domain Aβ antibody, as the capture antibody and HJ3.4 as the detection antibody (Supplementary Fig. 1B).

### Extraction of soluble Aβ aggregates

We tested a variety of single extraction, serial extraction and detergent-enhanced extraction methods. We determined that a single extraction by dounce homogenization in phosphate buffered saline (PBS) plus a sub-critical micelle concentration (0.45% weight/volume) of the zwitterionic detergent [(3-Cholamidopropyl)dimethylammonio]-1-Propanesulfonate (CHAPS) was appropriate. PBS + 0.45% CHAPS extraction yielded nearly 2-fold higher concentrations of soluble Aβ aggregates than extraction in PBS alone, and more than PBS + 4 other detergents at sub-critical micelle concentrations (Supplementary Fig. 2A). Furthermore, while a second extraction in PBS alone yielded 36% additional soluble Aβ aggregates after the first extraction, a second extraction in PBS+0.45% CHAPS yielded only 15% additional soluble Aβ aggregates and subsequent extractions yielded very little additional soluble Aβ aggregate, indicating efficient first pass extraction in PBS + 0.45% CHAPS (Supplementary Fig. 2B–D). Notably, even after 5 extractions in PBS + 0.45% CHAPS, additional Aβ could be extracted in 0.5 M Guanidine (Supplementary Fig. 2C). Taken together, these results were consistent with the material in the first PBS + 0.45% CHAPS extraction representing efficient extraction of a finite pool of soluble Aβ aggregates but not consistent with extraction of normally insoluble Aβ from plaques.

**Figure 2 f2:**
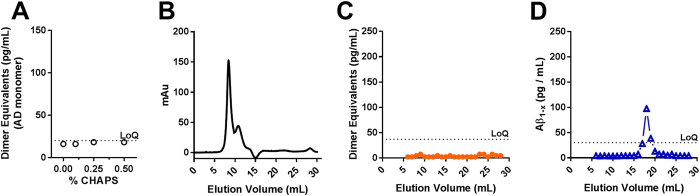
Sub-critical micelle concentration of CHAPS in PBS does not induce *ex vivo* aggregation of human AD brain derived Aβ monomers. (**A**) Titration of CHAPS during homogenization of normal control cortical tissue spiked with 2 ng/mL human AD brain-derived Aβ monomer. No induction of detectible Aβ aggregates. (**B**–**D**). Size exclusion chromatography of the 475,000× g RCF sucrose cushion fraction from normal control cortical tissue spiked with 2 ng/mL AD brain-derived Aβ monomer. (**B**) Total protein, (**C**) Soluble Aβ aggregate assay indicating no detectible Aβ aggregates, (**D**) Aβ_1−x_ assay indicating <10% of spiked monomer present in sucrose cushion (high molecular weight enriched) portion of the preparation. LoQ: limit of quantitation.

Importantly, extraction in PBS + 0.45% CHAPS did not result in *ex vivo* aggregation of monomeric Aβ. We isolated native monomeric Aβ from human Alzheimer’s disease (CDR3) brain tissue (Fig. 1), then added the AD-derived monomer (2 ng/mL) to the buffer during the homogenization of cognitively normal, AD pathology-negative human brain tissue in the presence of sub-critical micelle concentrations of CHAPS. The homogenates were centrifuged at 100,000× g prior to assessment using the dimer equivalent ELISA. We observed no ELISA signal at each concentration, indicating that 0.45% CHAPS did not induce *ex vivo* formation of soluble Aβ aggregates within the level of detection (Fig. 2A). Furthermore, there were no soluble Aβ aggregates detected after 475,000× g RCF ultracentrifugation (Fig. 2B–D). We repeated this control experiment using synthetic Aβ_1-42_ and found no effect of 0.45% CHAPS on soluble Aβ aggregate formation as a function of concentration of synthetic Aβ_1-42_ up to 20 ng/mL (Supplementary Fig. 3). In contrast, the commonly used detergent sodium dodecyl sulfate (SDS) dramatically enhanced *ex vivo* aggregation of Aβ at concentrations of 0.2% or higher (Supplementary Fig. 4), which may be relevant for interpretation of results from other groups that used this detergent in the context of polyacrylamide gel electrophoresis (see Supplementary Discussion). Triton X-100 also caused some *ex vivo* aggregation at 1%, but Tween-20 did not affect aggregation at concentrations up to 1% (Supplementary Fig. 4). In summary, we found that a single extraction in PBS + 0.45% CHAPS provided a high yield of soluble Aβ aggregates from human brain tissue without causing detectible *ex vivo* aggregation, but other detergents such as SDS and Triton X-100 appeared to cause *ex vivo* aggregation.

### Minimizing non-specific loss of Aβ

It became clear during our investigations of soluble Aβ aggregate extractions that the quantities of these aggregates were not stable over time; there was a rapid loss of Aβ aggregates, likely due to nonspecific binding to surfaces. Even low-protein binding tubes and tips did not eliminate this loss. We therefore modified our technique to include blocking of every tube and every pipet tip with a 1% bovine serum albumin (BSA) solution. With BSA blocking, non-specific loss was dramatically reduced both at room temperature and 4 °C (Supplementary Fig. 5). BSA blocking reduced loss of Aβ but had no apparent effect on the size exclusion chromatography profile of Aβ in human brain lysates (Supplementary Fig. 6). Without albumin blocking, we estimate that >90% of all Aβ aggregates would have been lost during even our most efficient purification protocols.

### Isolation of soluble Aβ aggregates from other forms of Aβ

For the second task, separating soluble Aβ aggregates from other forms of Aβ, we used a differential ultracentrifugation protocol. By definition, solubilizing the preparation in PBS separates ‘soluble’ forms of Aβ from ‘insoluble’ forms of Aβ. We pelleted the less soluble forms of Aβ by centrifugation at 100,000× g (Fig. 1). [Fig f2][Fig f3][Fig f4][Fig f5]Subsequent experiments demonstrated that the forms of Aβ in the 100,000× g pellet were morphologically distinct from the more soluble forms of Aβ present in the 100,000× g supernatant (see Fig. 6, below).

**Figure 3 f3:**
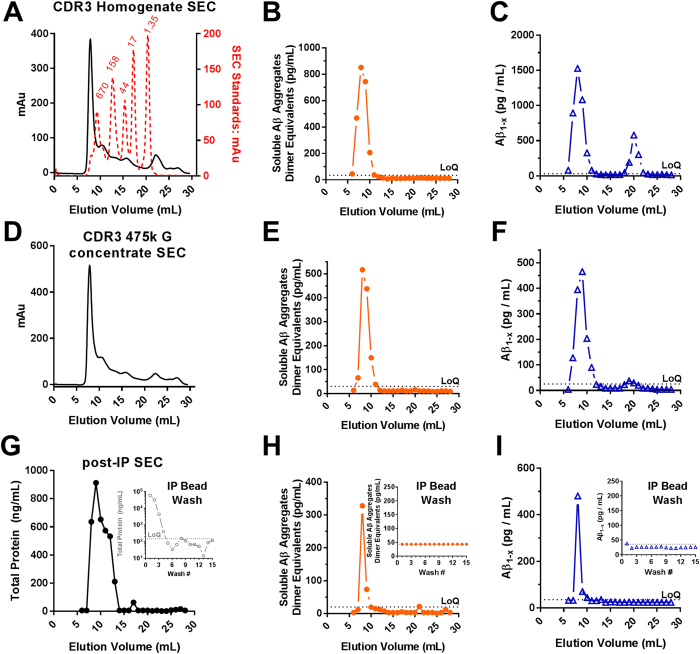
Enrichment and preservation of soluble high molecular weight Aβ aggregates from human AD brain following differential centrifugation and immunoprecipitation. (**A**) Size exclusion chromatography (SEC) total protein profile (measured by absorption) of a CDR3 tissue homogenate prepared in 1xPBS containing 0.45% CHAPS; overlay with Bio-Rad gel filtration standards (red) with molecular weight in kDa indicated (red text). (**B**,**C**). Soluble Aβ aggregate and Aβ_1−x_ assays on SEC fractions of original lysates. LoQ: limit of quantitation. (**D**) SEC total protein profile of the sucrose cushion fraction after ultracentrifugation at 475,000× g. (**E**,**F**) Soluble Aβ aggregate and Aβ_1−x_ assays on SEC fractions of sucrose cushion fraction after ultracentrifugation at 475,000× g, demonstrating the preservation of soluble Aβ aggregates and separation from monomers. (**G**) SEC total protein profile (measured by NanoOrange) of the 475,000× g sucrose cushion fraction followed by immunoprecipitation (IP), washing, and elution with ammonium hydroxide. (**H**,**I**) Soluble Aβ aggregate and Aβ_1−x_ assays on eluted high molecular weight soluble Aβ aggregates. *Insets*: Quantification of protein (note log scale in panel G), soluble Aβ aggregates, and Aβ_1−x_ in the immunoprecipitation bead washes.

**Figure 4 f4:**
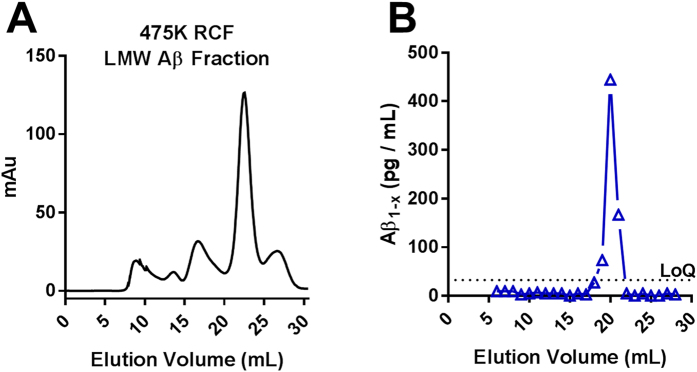
Aβ monomer is the predominant size form of Aβ from human AD brain in the top layer after 475,000× g ultracentrifugation. (**A**) SEC total protein profile from 1 mL of the top 5 mL supernatant layer removed following ultracentrifugation at 475,000× g. (**B**) Aβ_1−x_ assay on the same supernatant SEC fractions.

Previously^40^, we demonstrated using size exclusion chromatography that the soluble Aβ aggregates extracted from human brain detected by our assay were large, eluting close to the void volume in fractions 7–10 near the 670 kDa globular protein size standard on a Superdex 200 column (Fig. 3A–C). In contrast, monomeric Aβ is ~4 kDa in size, eluting in fractions 19–21. We therefore considered using size exclusion chromatography to separate soluble Aβ aggregates from monomers, but found that this method was inefficient for scale-up because size exclusion chromatography performed poorly for large liquid volumes. We lysed each gram of brain in 10 mL of buffer whereas only 1 mL at a time could be run on size exclusion chromatography. We next considered the use of molecular weight cutoff centrifuge filters, but found that there was substantial loss of soluble Aβ aggregates during this process and that it did not efficiently remove low molecular weight species from the retentate (Supplementary Fig. 7). Third, we considered ammonium sulfate precipitation of high molecular weight aggregates. This method scaled up well, did not result in substantial loss of soluble Aβ aggregates, and did not induce *ex vivo* aggregation of monomeric Aβ, but it did not fully separate soluble Aβ aggregates from endogenous monomeric Aβ (Supplementary Fig. 8). Finally, we turned to differential ultracentrifugation and found that 1 hour at 475,000× g kept monomeric Aβ in the upper phase and pelleted the soluble Aβ aggregates. Because the soluble Aβ aggregates were difficult to completely resuspend after pelleting at 475,000× g, we modified the 475,000× g ultracentrifugation protocol to include a 70% sucrose cushion, allowing nearly complete recovery of the soluble Aβ aggregates. The Aβ in the 475,000× g sucrose cushion was essentially all high molecular weight with nearly undetectable low molecular weight Aβ (Fig. 3D–F). In a complementary fashion, the Aβ in the 475,000× g supernatant fraction was entirely low molecular weight (Fig. 4). This method could be applied to up to 8 tubes at a time with 26 mL of material per tube. These results confirmed that differential ultracentrifugation was an effective and scalable method to separate soluble Aβ aggregates from other forms of Aβ.

### Separation of soluble Aβ aggregates from other proteins

For the third task, separating soluble Aβ aggregates from other proteins, we considered immunoprecipitation, ion exchange chromatography, and hydrophobicity chromatography; we settled on a dual antibody immunoprecipitation with ammonium hydroxide elution as the final method.

Ion exchange chromatography with strong anion and strong cation exchangers at neutral and basic pH’s were tested (acidic pH’s were avoided to prevent passing Aβ through its pKa at ~5.5). Anion exchange chromatography at pH 8 yielded a discrete soluble Aβ aggregate peak (Supplementary Fig. 9A,B) but only a 3.1 fold enrichment of soluble Aβ aggregates compared with total protein. The peak occurred over 14 mL, resulting in substantial dilution.

Hydrophobicity interaction chromatography with several resins (butyl sepharose, phenyl sepharose, and octyl sepharose) and several mobile phases (sodium chloride, ammonium sulfate, sodium sulfate) was tested. No clear enrichment of soluble Aβ aggregates was obtained, and the soluble Aβ aggregates eluted over a broad range of salt concentrations (Supplementary Fig. 9C,D).

Immunoprecipitation using beads conjugated with two different monoclonal antibodies recognizing two distinct epitopes was used to increase avidity. We used HJ3.4 recognizing the canonical free Aβ N-terminus, and HJ5.1 recognizing a mid-domain epitope conjugated directly to cyanogen bromide sepharose beads. Incubation of brain lysates overnight at 4 °C with these dual antibody conjugated beads resulted in a complete immunodepletion of Aβ; no detectible Aβ was present in the supernatant. Elution in the detergent lithium docecyl sulfate (LDS) at 95 °C dramatically altered the size forms of Aβ, elution in the high salt buffer 5 M lithium chloride resulted in incomplete recovery, and elution in acidic conditions with 200 mM glycine pH 2.7 resulted in almost no recovery (Supplementary Fig. 10A–C). In contrast, elution in 150 mM ammonium hydroxide pH 10.5 resulted in nearly complete recovery of Aβ without changing the size forms resolved on size exclusion chromatography (Supplementary Fig. 10D). Immunoprecipitation of the isolated high molecular weight soluble Aβ aggregates from the 475,000× g sucrose cushion with these dual antibody beads and elution with ammonium hydroxide resulted in a nearly complete recovery of Aβ, a sharpening of the Aβ peak into fractions 8–9, and no change in the size forms of Aβ present (Fig. 3G–I). Total protein fell ~1000 fold during the bead washes (Fig. 3G *inset*), but no detectible Aβ was lost in these washes (Fig. 3H,I *insets*). These results confirmed that dual antibody immunoprecipitation with ammonium hydroxide elution was an effective method for separating Aβ from other proteins.

### Quantitative bookkeeping

**Figure 5 f5:**
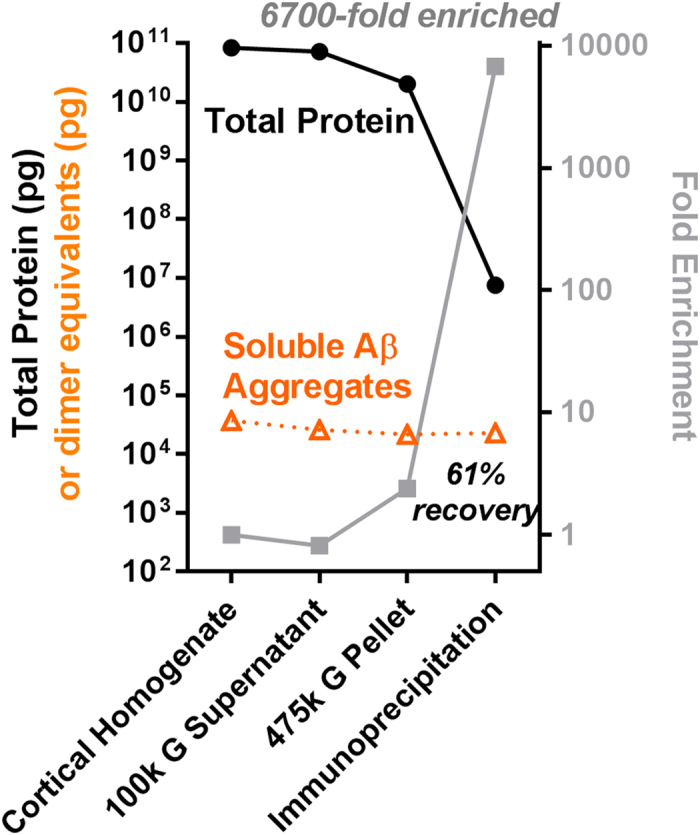
“Quantitative bookkeeping”. Total protein and soluble Aβ aggregate assays (note log scale) performed on each step of the isolation and purification of soluble Aβ aggregates from human AD brain.

We took aliquots from each step during our final purification protocol and measured soluble Aβ aggregate concentrations and total protein concentrations for the purposes of quantitative bookkeeping. We found that a typical purification protocol resulted in over 6000 fold purification of soluble Aβ aggregates relative to total protein and >60% recovery of the soluble Aβ aggregates present in the original brain lysate (Fig. 5). The recovery rate was calculated by measuring the concentration of soluble Aβ aggregates in the initial lysates and the concentration in the final immunoprecipitated and eluted material from the 475,000× g sucrose cushion phase. The calculations were corrected for 5–10 microliter aliquots of material taken for analysis at each step of the purification. The small quantities of soluble Aβ aggregates in the 100,000× g pellet and in the 475,000× g supernatant were considered part of the ~40% loss. In 6 distinct preparations from 6 different Alzheimer’s disease brain samples with extensive Aβ plaque pathology (Supplementary Fig. 11), we recovered approximately 2–6 ng dimer equivalents per gram of brain tissue and were able to enrich soluble Aβ aggregates to at least 0.1 to 0.6 percent of total protein (Supplementary Table 1). This enrichment may be a substantial underestimate; the actual purification may be substantially higher (see Discussion).

### Morphological characteristics of soluble Aβ aggregates

**Figure 6:**
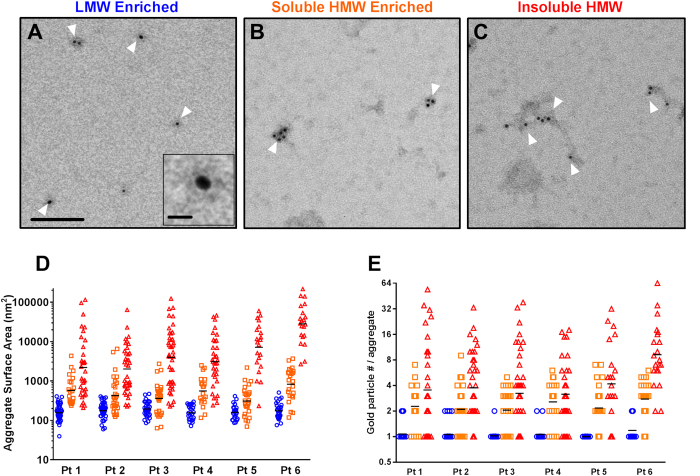
Immunoelectron microscopic analysis of soluble Aβ aggregates from human AD brain in comparison to low molecular weight Aβ and insoluble aggregates. Labeling was performed using the anti-Aβ antibody HJ3.4 followed by anti-mouse secondary conjugated to 6 nm diameter gold beads. (**A**) Exemplar immunoelectron microscopic images of low molecular weight (LMW) Aβ immunoprecipitated from the 475,000× g supernatant. White arrows indicate individually resolved objects with typically 1 but occasionally 2 gold beads. Scale bar = 100 nm. *Inset* shows a single 6 nm gold bead, with scale bar 10 nm. (**B**) Exemplar immunoelectron microscopic images of soluble high molecular weight (HMW) Aβ aggregates immunoprecipitated from the sucrose cushion after 475,000× g ultracentrifugation. Arrows indicate compact aggregates with multiple gold beads. (**C**) Exemplar immunoelectron microscopic images of insoluble high molecular weight Aβ aggregates immunoprecipitated from the pellet after 100,000× g ultracentrifugation. Arrows indicate elongated and irregular aggregates with multiple gold beads per aggregate. (**D**) Surface area measurements (in nm^2^, note log scale) of the gold labeled objects in the three fractions: LMW (blue circles), soluble HMW (orange squares), and insoluble (red triangles). Preparations were made from 6 AD patients; 10 random fields per patient were analyzed in an automated fashion. (**E**). Number of gold particles per aggregate were counted in an automated fashion from the same images.

We used immunoelectron microscopy (EM) to directly characterize the size and number of Aβ immunoreactive sites per aggregate (Fig. 6). The N-terminal specific antibody HJ3.4 was used for these analyses, followed by secondary antibodies conjugated to 6 nm gold beads. The three differential ultracentrifugation separated fractions of Aβ were morphologically distinct. The material immunoprecipitated from the low molecular weight (475,000× g supernatant) fraction consisted of ovoid structures (Fig. 6A), on average ~200 nm in surface area (Fig. 6D), which bound typically exactly 1 but occasionally 2 anti-Aβ antibodies, (Fig. 6E). The material immunoprecipitated from the high molecular weight soluble Aβ aggregate (475,000× g sucrose cushion) fraction consisted of larger irregular structures (Fig. 6B), with median size ~700 nm^2^ in surface area which bound typically 2-3 anti-Aβ antibodies (range 1–9). The material immunoprecipitated from the relatively insoluble high molecular weight (100,000× g pellet) fraction consisted of even larger and more elongated structures (Fig. 6C), with median size 2000–29,000 nm^2^ in surface area and a wide range of anti-Aβ antibody binding stoichiometries. Some of the larger aggregates from the insoluble fraction had irregular fibrillar mesh-like structures (Supplementary Fig. 12), whereas fibrillar structures were never observed in the human brain soluble Aβ aggregate fractions.

**Figure 7:**
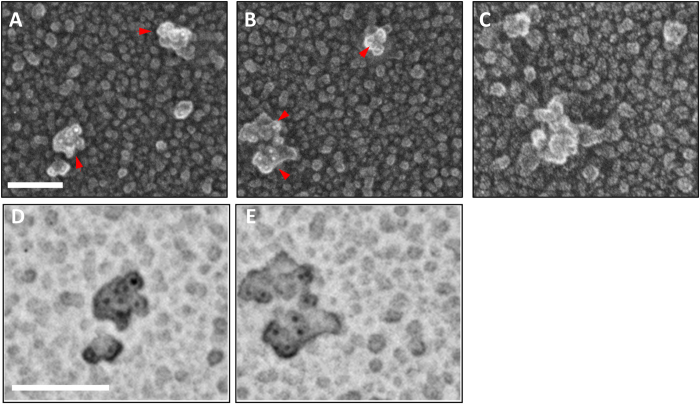
Quick-freeze deep etch negative replica immunoelectron microscopic images of soluble Aβ aggregates from human AD brain. (**A**,**B**) Exemplar images of soluble high molecular weight Aβ aggregates immunoprecipitated from the sucrose cushion after 475,000× g ultracentrifugation spotted onto glass and stained with N-terminal Aβ antibody HJ3.4 followed by anti-mouse secondary conjugated to 6 nm diameter gold beads. Replicas were produced by platinum deposition and mounted for imaging. Red arrows indicate aggregates with multiple gold bead (white) labeling. Scale bars 100 nm: (**C**) Images with no primary antibody indicating absence of nonspecific binding of gold-labeled secondary antibody. (**D**,**E**) Expanded view of aggregates from panels A and B with contrast inverted to make the gold bead labels (black) more apparent.

Quick-freeze deep etch immuno-EM further revealed that the soluble Aβ aggregates consisted of clusters of approximately 10 × 20 nm ovoid substructures, with each ovoid substructure appearing to bind at most 1 anti-Aβ antibody (Fig. 7 and Supplementary Fig. 12). Quick-freeze deep etch methods were useful for morphology but not optimal for automated quantitative analysis because the intensity of the edges was often similar to the intensity of the gold beads.

There were modest but statistically significant correlations between the area of the soluble high molecular weight Aβ aggregates and the number of anti-Aβ antibodies bound (Supplementary Fig. 13). Based on the scatter plots of area vs. number of anti-Aβ antibodies bound, there appeared to be two different classes of soluble Aβ aggregates; those for which there was a very strong linear relationship between area and antibody binding stoichiometry with approximately 200 nm^2^ per antibody binding, and those for which the area exceeded that which could be accounted for by antibody binding stoichiometry. In this second class, a large portion of the surfaces of the soluble Aβ aggregates were not immunoreactive with the HJ3.4 antibody. This could indicate additional constituents other than HJ3.4 immunoreactive Aβ. The correlation between area of the relatively insoluble high molecular weight aggregates and the number of anti-Aβ antibodies bound was very tight (Supplementary Fig. 14), as would be expected for a single class of aggregates. There was also a large portion of the surfaces of the relatively insoluble aggregates that was not immunoreactive with the HJ3.4 antibody. Synthetic Aβ_1-40_ aggregates were more densely labeled by HJ3.4, but still not completely saturated with gold particles (Supplementary Fig. 12).

### Mass spectrometric analysis of soluble Aβ aggregates

**Figure 8:**
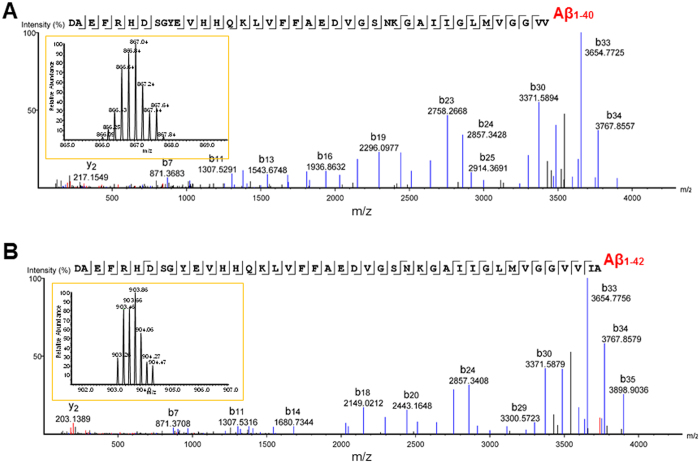
Mass spectrometry of soluble Aβ aggregates from human AD brain. (**A**) Liquid chromatography-tandem mass spectrometry (LC-MS/MS) spectrum for undigested, full-length Aβ_1-40_. Each peak represents an ion fragmented from Aβ_1-40_, with peaks labeled ‘*b*’ representing N-terminal fragment ions and peaks labeled ‘*y*’ representing C-terminal fragment ions. The numbers indicate measured mass/charge ratio (*m*/*z*). The single letter amino acid code across the top indicates the *de novo* sequence identified by mass spectrometry, which matches the amyloid precursor protein sequence corresponding to Aβ_1-40_. The line breaks between amino acids indicate a cleavage of the amide bond between two adjacent amino acids producing fragment ions. The lines below each amino acid indicate a detected ‘*b*’ ion, and lines above indicate a detected ‘*y*’ ion. *Inset:* isotopic envelope for the +5 charged, full-length Aβ_1-40_: the peaks are spaced 0.2 daltons apart at *z* = +5 because the naturally occurring isotopes (e.g. ^13^C and ^15^N) differ by 1 dalton. For the +5 ion, the observed *m/z* was 866.4351 (theoretical *m*/*z* = 866.4370), which was −2.1 parts per million (ppm) error from the theoretical mass of Aβ_1-40_. (**B**) Spectrum for full length Aβ_1-42_. For the +5 ion, the observed *m/z* was 903.2623 (theoretical *m*/*z* = 903.2612), which was 1.2 ppm error from the theoretical mass of Aβ_1-42._

We performed top-down tandem mass spectrometry on the soluble Aβ aggregates isolated from human AD brain and detected full length (undigested) Aβ_1-40_ and Aβ_1-42_ (Fig. 8 and Supplementary Fig. 15). The mass spectrometry results were very clean, with clear peaks within the isotopic envelope spaced every 0.2 daltons, indicating a +5 charge state. A very large number of fragmented ‘*b*’ ions and ‘*y*’ ions were detected, yielding a high degree of confidence in the identity of the sequences. These results confirm that the soluble Aβ aggregate preparations from human AD brain truly contain Aβ. Many other forms of Aβ were also detected (see below.)

## Discussion

In summary we have developed a method for reliably enriching soluble Aβ aggregates from human brain tissue, separating them from other forms of Aβ, and purifying them more than 6000 fold with less than 40% loss of starting material. These soluble Aβ aggregates are believed to be play a critical role in neurodegeneration relating to dementia of the Alzheimer’s type, however they had not been previously isolated from human brain and purified sufficiently for detailed analysis. Our isolation and partial purification was sufficient for immunoelectron microscopic characterization, which indicated that these soluble Aβ aggregates appear to consist of clusters of ovoid, non-fibrillar structures typically with 2–3 binding sites for antibodies binding amino-terminal Aβ.

We are in the early stages of fully characterizing the specific Aβ proteoforms present in the soluble Aβ aggregates using top-down (undigested) tandem mass spectrometry. Absolute quantification of the abundance of various forms of Aβ in the soluble Aβ aggregates has not yet been performed, but is an important area of ongoing investigation. A full characterization of the Aβ proteoforms and possible associated proteins is beyond the scope of this communication.

### Relationship to Previous Studies

The relationship between our findings and those of previous research groups investigating soluble Aβ aggregates in human AD brain is complex (please see Supplementary Table 2 and Supplementary Discussion), but may be explained largely by differences in experimental methods. Our results are most similar to those of Noguchi *et al*., who reported high molecular weight globular assemblies of Aβ from Alzheimer’s disease human brain after saline-based homogenization^24^. In contrast, other investigators have reported smaller forms of soluble Aβ assemblies^20^,^23^, or assessed partially dissociated aggregates likely derived from water insoluble plaques^41^. Many of the previously reported preparations have involved SDS PAGE to assess the size forms, which can both break apart larger Aβ assemblies and cause monomeric Aβ to aggregate *ex vivo*^42^. A highly cited previous report^23^ used size exclusion chromatography to assess the size of the soluble Aβ aggregates, but the running buffer was ammonium acetate pH 8.5 which can cause changes in assembly of protein complexes^43^,^44^,^45^,^46^,^47^. We found that ammonium acetate pH 8.5 consistently changes the apparent size of soluble Aβ aggregates (Supplementary Fig. 16), though another recent report using a different assay indicates that it may not always do so^35^. Furthermore, detection of the putative Aβ assemblies often involved antibodies which recognize epitopes present in amyloid precursor protein as well as Aβ, such that assemblies of Aβ cannot be distinguished unambiguously from fragments of amyloid precursor protein. Thus, although several groups have reported characteristics of soluble human brain Aβ aggregates that differ from those described here, we have demonstrated through a careful series of control experiments that our method minimizes many previously concerning potential sources of *ex vivo* aggregation and de-aggregation.

### Limitations and Future Directions

The concentrations of soluble Aβ aggregates measured here may be underestimates, since our ELISA detects only species with at least 2 free canonical Aβ N-termini (amino acids DAEFR…). Our initial data from mass spectrometric analysis indicate multiple other forms of Aβ with truncated and post-translationally modified N-termini^48^. It is therefore likely that our enrichment of Aβ may be quantitatively greater than reported. For the same reason, the number of Aβ peptides per aggregate may also be underestimates. Future additional immunoelectron microscopy studies using antibodies recognizing different Aβ species will be of interest.

Because the antibody HJ3.4 could also in theory recognize the C99 peptide or C-terminally truncated versions of this peptide, we cannot be absolutely certain that all of the detected particles represent Aβ. Our mass spectrometry results, however, have not yet revealed any peptides longer than 43 amino acids (Wildburger *et al*., *in preparation*).

It is likely that there are other proteins associated with the soluble Aβ aggregates characterized here; future investigations involving discovery based mass-spectrometry and double immunoelectron microscopy validation are underway, but are beyond the scope of this report.

We cannot be certain that the sizes of the aggregates and numbers of Aβ binding sites per aggregates represent the state of these species in the living human brain. It is possible that post-mortem events such as autolysis could have altered these aggregates. Experiments are underway to assess the effects of varying simulated post-mortem intervals in transgenic mice. Methods to assess soluble Aβ aggregates in the living human brain are still under development. The relationship between soluble Aβ aggregates in the brain and in the cerebrospinal fluid is likely to be complex. To date, we and others have not been able to detect soluble Aβ aggregates in human cerebrospinal fluid^40^,^49^, though some groups have done so^50^,^51^,^52^. Assessments of brain biopsy tissue from patients undergoing normal pressure hydrocephalus surgery, nearly half of whom may also have Aβ pathology^53^, represent a logical direction for addressing potential post-mortem artifacts. Direct detection in the brain using molecularly specific electrode technology may represent a novel approach^54^.

We also cannot be certain of whether changes in the properties of the soluble Aβ aggregates occurred during purification. The consistent size profile of the aggregates from raw lysates to finale eluates following immunoprecipitation indicate that there were no gross changes, but more subtle effects such as loss of low molecular weight components cannot be ruled out. Likewise, the addition of exogenous monomeric Aβ as a control for artifactual aggregation would not be expected to mimic effects on intracellular Aβ. Selective binding to EM grids could also have biased our observations. We cannot fully exclude a shift in apparent size of soluble Aβ aggregates due to interaction with the matrix during SEC. However, synthetic Aβ monomer and synthetic Aβ dimer run true to size under identical SEC conditions^40^. Furthermore, the large size observed by SEC is generally concordant with the immuno-EM observations, though at present it is not possible to directly estimate the molecular weight of the soluble Aβ aggregates from the EM observations. A future direction will involve orthogonal biophysical characterization of the aggregates from raw lysates vs. final eluates based on charge (ion exchange) and hydrophobicity (HIC). The quantities of soluble Aβ aggregates we have extracted are as yet too low to allow dynamic light scattering, circular dichroism, or fast photochemical oxidation of protein- mass spectrometry.

The Aβ species described here may be just one of many classes of soluble Aβ aggregates. Other classes of aggregates that do not bind HJ3.4 would not be detected or characterized. Thus, a priority for future research will be a broader exploration of the spectrum of Aβ aggregates present in the human AD brain using other detection reagents (see Supplementary Discussion).

Importantly, the physiological effects of the soluble Aβ aggregates isolated and partially purified using these methods has not been characterized. In previous studies, low molecular weight soluble Aβ aggregates derived from human brain potently impaired synaptic plasticity in rodent brain slices^23^ and caused tau hyperphosphorylation with morphological disruption in cultured neurons^27^. However, it was reported that the high molecular weight soluble Aβ aggregates after size exclusion chromatography in ammonium acetate did not affect synaptic plasticity^23^. High molecular weight human brain aggregates separated in saline have been reported to cause toxicity in cultured neurons^24^. The relationship between these relatively acute *in vitro* physiological effects and slowly progressive dementia of the Alzheimer’s type has yet to be determined. Underscoring the relevance of this line of investigation is the finding that the levels of soluble Aβ aggregates in lysates from human brain were strongly related to dementia status, and fully differentiated demented from non-demented individuals after normalizing for Aβ plaque burden^40^.

### Implications

We anticipate that the method presented here for extracting, isolating, and purifying soluble Aβ aggregates will facilitate many additional investigations of the properties of these aggregates. One of the major long-term goals of this line of investigation is to discover the distinguishing structural characteristics of the most relevant forms of soluble Aβ aggregates from the human brain. The specific details of their molecular composition will be of great interest with regard to developing specific candidate therapeutics to prevent their formation, enhance their clearance, or block their effects. Direct measurements of these soluble Aβ aggregates in living humans using biofluids or by molecular imaging could then be used for pharmacodynamic characterization of candidate therapeutics. Importantly, the extent to which the soluble Aβ aggregates present in the human brain is similar to or different from the aggregates present in animal models of AD-like pathology is entirely unknown, but critical for the field of therapeutic development. If one or more animal models faithfully recapitulates the soluble Aβ aggregates present in humans, it would seem logical to use these models for therapeutic development rather than other models that do not accurately recapitulate the human-like aggregates. Thus, characterizing a range of animal models of AD-like pathology is a top priority. It is possible, however, that none of the existing animal models accurately recapitulate human soluble Aβ aggregates, and that new model systems will need to be developed^55^.

Furthermore, the purification scheme developed here may be thought of as a generalizable framework for assessment of proteins aggregates relevant to human neurodegenerative diseases. Soluble aggregates of tau, alpha synuclein, prion protein, TAR DNA-binding protein 43 (TDP43), fused in sarcoma (FUS), superoxide dismutase 1, huntintin, and others have been implicated in mediation of neurotoxic effects^56^ and are therefore potentially relevant therapeutic targets but have not been characterized in human brain tissue.

## Materials and Methods

### Regulatory Compliance

All protocols were carried out in accordance with the Charles F. and Joanne Knight Alzheimer’s Disease Research Center and Washington University guidelines. This specific study was approved by the Knight Alzheimer’s Disease Research Center tissue committee. All donors or their surrogates gave informed consent for their brains to be used for research studies.

### Homogenization of human cortical tissue

Human frontal cortical tissue samples were obtained from the Knight Alzheimer’s Disease Research Center at Washington University School of Medicine in Saint Louis, Missouri. Cognitive status was determined with a validated retrospective postmortem interview with an informant to establish the Clinical Dementia Rating (CDR). Pathologically confirmed CDR3 cases were selected (n = 6, 84.1+/−6.6 years at death, 11.4+/−4.8 hours post mortem interval, 4 female & 2 male). In addition, two cognitively normal (CDR0, negative AD pathology) cases were selected for use in control experiments (n = 2). Frozen cortical samples were weighed and placed into ice-cold Phosphate-Buffered Saline (PBS) containing protease inhibitors (137 mM sodium chloride, 7.76 mM sodium phosphate dibasic, 2.17 mM monopotassium phosphate, 2.7 mM potassium chloride, 2 μg/mL aprotinin, 1 μg/mL leupeptin) followed by removal of leptomeninges and apparent vasculature. The tissue was then immediately dounce homogenized in ice-cold PBS containing protease inhibitors at a 10:1 PBS volume:tissue weight ratio using a Potter-Elvehjem Teflon coated tissue grinder at a constant 25 manual strokes. The addition of 3-[(3-Cholamidopropyl)dimethylammonio]-1-Propanesulfonate (CHAPS) at a final concentration of 0.45% (w/v) was performed for experiments as detailed below.

### Measurement of soluble Aβ aggregates

We adapted our previously developed^40^ “dimer equivalents” ELISA assay to a 96-well format for the quantitation of soluble Aβ aggregates during purification. The mouse monoclonal antibody HJ3.4 was coated to 96-well Nunc Maxisorp plates (#464718, Nalge Nunc, Rochester, NY) at 20 μg/mL in carbonate buffer (35 mM sodium bicarbonate, 16 mM sodium carbonate, 3 mM sodium azide, pH 9.6) in 100 μl/well overnight at 4 °C. Plates were washed 5x between steps with PBS using a BioTek EXL405 plate washer (BioTek, Winooski, VT). The assay plates were blocked using 0.2 μm filtered 4% BSA (#7030, Sigma-Aldrich, St. Louis, MO) in PBS for 1 hour at room temperature. Samples or dimer standard were added neat or diluted in standard diluent (0.2 μm filtered 0.25% BSA, 0.005% Tween-20, 300 mM Tris, 3 mM sodium azide, 2 μg/mL aprotinin, 1 μg/mL leupeptin, in PBS) to 100 μL final volume and loaded. An 8-point standard curve was generated using 2000, 1000, 500, 250, 125, 62.5, and 31.25 pg/mL of Aβ_1-40Ser26Cys_ dimer in DMSO (AnaSpec, Fremont, CA) diluted in standard diluent and loaded in triplicate in all experiments. All samples and standard were kept on ice during handling. Assay plates were incubated at 4 °C overnight. Biotinylated HJ3.4 at 100 ng/mL was then added in PBS containing 0.2 μm filtered 0.5% BSA plus 0.005% Tween-20 for 1 hour at room temperature with gentle agitation. Poly-streptavidin HRP-20 (65R-S103PHRP, Fitzgerald, Acton, MA) was then incubated at 30 ng/mL in PBS containing 0.2 μm filtered 0.5% BSA plus 0.005% Tween-20 for 1 hour at room temperature with gentle agitation. After a final wash, the assay was developed by addition of 3,3′,5,5′-Tetramethylbenzidine (T5569, Sigma-Aldrich) and the absorbance was read on a BioTek Synergy 2 plate reader at 650 nm.

### Measurement of Aβ_1−x_ isoforms

Assessment of isoforms of Aβ starting with amino acid 1 and ending after the mid-domain epitope of the capture antibody (approximately amino acid 28), here termed Aβ_1−x_, in the purification steps was determined by ELISA using the mid-domain HJ5.1 to capture and the N-terminal specific HJ3.4 to detect bound peptide. HJ5.1 was coated to 96-well Nunc Maxisorp plates at 20 μg/mL in carbonate buffer (35 mM sodium bicarbonate, 16 mM sodium carbonate, 3 mM sodium azide, pH 9.6) in 100 μ/well overnight at 4 °C. Plates were washed 5x between steps with PBS using a BioTek EXL405 plate washer. The assay plates were blocked using 0.2 μm filtered 4% BSA in PBS for 1 hour at room temperature. Samples and standard were diluted in standard diluent (0.2 μm filtered 0.25% BSA, 0.5 M guanidine-HCl, 0.005% Tween-20, 300 mM Tris, 3 mM sodium azide, 2 μg/mL aprotinin, 1 μg/mL leupeptin, in PBS) to a 100 μL volume and loaded. An 8-point standard curve was generated using 2000, 1000, 500, 250, 125, 62.5, and 31.25 pg/mL of Aβ_1-40_ synthetic monomer peptide (AnaSpec) and loaded in triplicate in all experiments. All samples and standard were kept on ice during handling. Assay plates were incubated at 4 °C overnight. Development of the assay was identical to the soluble Aβ aggregate assay as described above.

### Assessment of other detergents for extraction of soluble Aβ aggregates

To determine the potential utility of common biochemical detergents for increased Aβ recovery we homogenized tissue in the presence of sub-critical micelle concentration detergent in PBS with protease inhibitor. The following concentrations of each detergent were used: sodium dodecyl sulfate (0.2% w/v), Triton™ X100 (0.018% v/v), Tween^®^ 20 (0.007% v/v), 6-O-(N-Heptylcarbamoyl)-methyl-α-D-glucopyranoside (Hecameg, 0.6% w/v), *n*-Octyl glucoside (0.55% w/v), CHAPS (0.45% w/v). For each tissue sample, the material was finely diced prior to homogenization to ensure each detergent received approximately similar quantities of input. Following centrifugation at 100,000× g RCF, the homogenates were diluted and the amount of soluble Aβ aggregate determined by ELISA.

### Size exclusion chromatography

Analysis of the apparent molecular weight of the Aβ aggregates was achieved using size exclusion chromatography, which unlike most gel electrophoresis methods, does not require SDS or similar detergents. A maximum of 1 mL of enriched aggregate was injected onto a Superdex 200 10/300 GL column attached to an AKTA Purifier FPLC (GE Healthcare) and eluted with PBS containing 0.2 μm filtered 0.05% BSA at a flow rate of 0.5 mL/min. A total of twenty-three 1 mL elution fractions were collected starting from the 6th mL. The eluted volume was collected in 0.2 μm filtered 1% BSA blocked low-binding microcentrifuge tubes prior to further purification or analysis. Under identical conditions, we ran globular protein size standards (Biorad) included thyroglobulin (670 kDa), gamma-globulin (158 kDa), ovalbumin (44 kDa), myoglobin (17 kDa), and vitamin B12 (1.35 kDa).

### Controls for *ex vivo* Aβ aggregation during CHAPS homogenization

To determine whether the addition of CHAPS to the purification method induces *ex vivo* aggregation, CDR3 derived monomer was spiked into cognitively normal human tissue during homogenization containing CHAPS at or below the critical micelle concentration. In addition, synthetic Aβ_1-42_ was similarly spiked into cognitively normal human tissue during homogenization containing CHAPS at 0.45% v/w in PBS. The homogenate was immediately centrifuged at 100,000× g RCF for 1 hour and the resulting supernatant applied to the soluble Aβ aggregate ELISA to measure the presence of any aggregates formed *ex vivo*. Furthermore, a 1 gram sample of cognitively normal cortex was homogenized with 0.45% CHAPS-PBS spiked with 2 ng/mL AD derived monomer. The homogenate was processed using the differential centrifugation protocol as described above. The resulting concentrate was separated by size exclusion chromatography on the Superdex 200 10/300 GL column and the presence or absence of soluble Aβ aggregates was assessed in the collected fractions.

### Control for *ex vivo* Aβ aggregation in other detergents

To determine if the typical concentrations of Triton X-100 (0.1–1.0%), sodium dodecyl sulfate (0.1–2.0%), and Tween20 (0.1–1.0%) results in *ex vivo* aggregation, we spiked 2 ng of AD-derived Aβ monomer at 2 ng/mL into homogenization buffer with the appropriate amount of cortical tissue from a cognitively normal, AD pathology negative brain. Following homogenization and centrifugation at 100,000× g RCF, the supernatant was loaded onto the soluble Aβ aggregate ELISA and developed as described.

### Reducing Aβ loss by blocking with bovine serum albumin

Non-specific loss of Aβ during multiple procedural steps is an additive problem. A significant reduction in nonspecific loss was achieved by incubation of every sample tube and micropipette tips with a solution of 0.2 μm filtered 1% BSA in PBS. Excess BSA was removed by washing with molecular grade water and then the plasticware was dried prior to use. In addition, during size exclusion chromatography we utilized 0.2 μm filtered 0.05% BSA in the mobile phase to reducing nonspecific loss to the Superdex 200 chromatography resin.

### Concentration by Macrosep concentrator

High molecular weight Aβ aggregates were concentrated using a 100 kDa molecular weight cut off centrifugal concentrator (#MAP100C36, Pall Corporation, Port Washington, NY). Freshly prepared cortical homogenate was clarified at 100,000× g RCF and transferred to a chilled 100 kDa molecular weight cut off concentrator. The concentrator was centrifuged at 4000× g RCF at 4 °C until the sample was concentrated approximately 10-fold. The concentrations of soluble Aβ aggregates were determined by ELISA.

### Ammonium sulfate precipitation

Owing to the high molecular weight nature of soluble Aβ aggregates they can be readily precipitated by addition of ammonium sulfate. Over a period of 30 minutes an ice-cold saturated solution of ammonium sulfate was added to the 100,000× g spun homogenate to a final concentration of 35% and then incubated on wet ice with gentle mixing for an additional 30 minutes. The precipitation was transferred to a series of 30 mL polypropylene centrifuge tubes and spun at 30,000× g for 30 minutes in a Sorvall RC5B Plus centrifuge using a SS-34 rotor operated at 4 °C. The resulting pellet was gently dissolved in ice-cold PBS containing protease inhibitors prior to further purification methods. To determine whether the process of ammonium sulfate precipitation induces *ex vivo* aggregation we performed a spike recovery assessment. Briefly, approximately 1 gram of cortical tissue from a cognitively normal participant was homogenized as described above with the addition of 2 ng/mL AD derived monomer. The homogenate was precipitated as described above and then separated by size exclusion chromatography before being assessed for Aβ_1−x_ and soluble Aβ aggregates by ELISA.

### Differential ultracentrifugation for isolation of soluble Aβ aggregates from other forms of Aβ

The cortical homogenates were transferred to chilled 30 mL polypropylene centrifuge tubes (#3119-0030, Nalgene) and spun at 17,000× g for 30 minutes in a Sorvall RC5B Plus centrifuge using a SS-34 rotor at 4 °C. The supernatant was transferred to 26.3 mL polycarbonate tubes (#355618, Beckman Coulter) and spun at 100,000× g for 1 hour in a Beckman Optima XPN-100 ultracentifuge using a type 70-Ti rotor operated at 4 °C under vacuum. The debris pellet from the 100,000× g spin was reserved for further analysis. The resulting 100,000× g spin supernatant was immediately transferred to a new 26.3 mL tube and underlayed with 1 mL of sterile 70% sucrose. The centrifuge tube was spun at 475,000× g for 1 hour in the Beckman Optima XPN-100 with a brake setting of “2” to reduce disturbance of the resulting protein layers. The supernatant was carefully removed in 5 mL layers and then a final 2 mL layer containing the high-molecular weight soluble Aβ concentrate overlaying the sucrose cushion was removed.

### Ion Exchange Chromatography (IEX)

The binding capacity and the elution resolution of Aβ species were assessed using anion exchange chromatography. Attached to the AKTA Purifier, 4.7 mL HiScreen strong anion (Q FF, #17-5053-01, GE Healthcare) column was equilibrated with 10 mM Tris, pH 8.0 prior to sample injection. A sample volume of 1 mL of cortical homogenate, diluted 1:5 with 10 mM Tris pH 8.0 (Buffer A) to reduce sodium chloride concentration, was injected onto the column. Following sample flowthrough, a gradient was performed with 10 mM Tris, pH 8.0, 3 M sodium chloride (Buffer B). Fractions were collected during the full gradient and assessed for soluble Aβ aggregates to measure the overall binding and elution.

### Hydrophobicity Interaction Chromatography (HIC)

The binding capacity and the elution resolution of Aβ species were assessed using HIC. Attached to the AKTA Purifier, a 4.7 mL HiScreen Butyl FF (#17-1357-01, GE) column was equilibrated with 10 mM Tris, pH 8.0, 3 M sodium chloride prior to sample injection. A sample volume of 1 mL of cortical homogenate, diluted 1:5 with 10 mM Tris pH 8.0, 3 M sodium chloride (Buffer A) to increase the sodium chloride concentration, was injected onto the column. Following sample flow through, a gradient was performed with 10 mM Tris, pH 8.0 (Buffer B). Fractions were collected during the full gradient and assessed for soluble Aβ aggregates to measure the overall binding and elution.

### Conjugation of monoclonal antibodies to cyanogen bromide (CNBr) sepharose

Immunoprecipitation of Aβ was performed using a combination of the N-terminal specific monoclonal, HJ3.4^57^, and a mid-domain specific monoclonal, HJ5.1^58^, conjugated to CNBr-Sepharose beads. Purified monoclonals were dialyzed against coupling buffer (0.1 M sodium carbonate, 0.5 M sodium chloride, pH 8.3) and then spun at 17,000× g in a table top refrigerated centrifuge to remove aggregated antibody complexes. One gram of activated CNBr-Sepharose was combined with a 6 mg equal mixture of each of the monoclonal antibodies in coupling buffer and incubated for 2 hours with gentle mixing. Unoccupied binding sites were blocked for 1 hour with 1 M ethanolamine, pH 8.0. The beads were rinsed with three cycles of 0.1 M sodium acetate, 0.5 M sodium chloride, pH 4.0 and coupling buffer to remove residual uncoupled protein. The beads were then washed and stored in PBS containing 0.02% sodium azide at 4 °C. All conjugation steps were performed at room temperature.

### Immunoprecipitation (IP) of Aβ

Following isolation, Aβ was immunopurified by addition of 100 μl dual antibody conjugated CNBr-Sepharose bead slurry per milliliter of concentrate in a preblocked microcentrifuge tube. Immunoprecipitation was allowed to occur overnight with gentle rotation (4 rpm) at 4 °C. The beads were isolated by centrifugation (2000× g, 2 min), and the supernatant was removed and saved for analysis. The beads were washed 15 times with 1 mL ice-cold PBS and gentle rotation for 2 minutes per wash. For assessment of total protein in each wash step the beads were washed with 0.1x PBS buffer, due to sodium chloride sensitivity with the NanoOrange assay. Bound Aβ was eluted by three rounds of 100 μl of 150 mM ammonium hydroxide and agitation for 5 minutes each. The elution volumes were pooled into a BSA blocked microcentrifuge tube and neutralized with 30 μl 3 M Tris, pH 7.2. Aliquots of the elution were removed for quantification of total protein, Aβ_1−x_, and soluble Aβ aggregates. Aβ eluates were stored at −80 °C or used immediately for electron microscopy.

### Alternative IP elution conditions

IP was performed on a freshly prepared cortical homogenate clarified at 100,000× g RCF as described above. The total IP bead volume was divided into four separate tubes and then 300 μL of either 5 M lithium chloride, 150 mM ammonium hydroxide pH 10.5, 200 mM glycine pH 2.7, or 1x NuPAGE LDS buffer (#NP0008, Invitrogen) was added to the respective tube and allowed to incubate at room temperature, or 95 °C for the LDS buffer, for 15 minutes. The resulting eluent was removed and immediately run over the Superdex 200 10/300 column and fractions collected. The distribution of Aβ following elution was assessed by indirect ELISA using the mid-domain specific mAb HJ5.1.

### Quantification of protein during purification steps

Total protein during each step of the purification procedure was quantitatively tracked by measurement with a colorimetric bicinchoninic acid (BCA) assay (#23225, ThermoFisher Scientific) using a reference BSA as the standard. Appropriate dilutions for each sample and the standard were combined with the assay reagent in a 96-well microplate and measured by absorbance at 562 nm on the BioTek plate reader. Because the protein levels were below the limit of sensitivity for BCA, total protein quantification of the immunoprecipitation wash steps and the resulting eluate were measured using a fluorescence-based NanoOrange assay (#N6666, Thermofisher Scientific) using a reference BSA as the standard. The lower limit of quantification for the NanoOrange assay is 150 ng/mL. Appropriate dilutions for each sample and the standard were combined with the assay reagent in a 96-well microplate and measured by fluorescence by excitation at 485 nm and emission at 590 nm on the BioTek plate reader. The NanoOrange assay produces interpretable results at sodium chloride concentrations of <30 mM.

### Immunoelectron microscopy

For quantification of purified amyloid aggregates, samples were absorbed onto glow-discharged carbon-coated 200-mesh Formvar grids (FCF200-Cu-UA, Electron Microscopy Sciences) by incubation of a 5 μL volume for two minutes. Sample grids were quenched for 30 minutes with quench buffer (50 mM lysine, 50 mM glycine, 50 mM ammonium chloride) followed by blocking for 30 minutes with 1% bovine serum albumin in NaHCa buffer (100 mM sodium chloride, 30 mM HEPES, 2 mM calcium chloride, pH 7). Sample grids were then incubated with HJ3.4 (1 μg/mL) in blocking solution for 1 hour. Following three washes with blocking solution, the grids were incubated with anti-mouse IgG conjugated to 6 nm colloidal gold (806.022, Aurion) diluted 1:15 in blocking solution. The grids were washed three times with NaHCa buffer followed by fixation in 2.5% glutaraldehyde for five minutes. The grids were negatively stained with a 1% (w/v) uranyl acetate solution and allowed to dry prior to imaging. All labeling steps were performed at room temperature. Images were collected using a JEOL JEM-1400 transmission electron microscope operating at 80 kV attached to an AMT XR111 high-speed pixel phosphor-scintillated 12-bit CCD camera.

Qualitative imaging using quick-freeze deep etch electron microscopy was performed according to published protocol, with minor modifications^59^. Samples were applied to acid cleaned glass chips and immunostained as described as above. Coverslips were rinsed in dH_2_O and frozen by abrupt application of the sample against a liquid helium cooled copper block with a Cryopress freezing machine. Frozen samples were transferred to a liquid nitrogen cooled Balzers 400 vacuum evaporator, etched for 20 minutes at −80 °C and rotary replicated with ~ 2 nm platinum deposited from a 15° angle above the horizontal, followed by an immediate ~10 nm stabilization film of pure carbon deposited from an 85° angle. Replicas were floated onto a dish of concentrated hydrofluoric acid and transferred through several rinses of dH_2_0 with a loopful of Photo-flo, picked up on formvar coated grids, and imaged on the JEOL JEM-1400 transmission electron microscope.

### FIJI based quantification of immunoelectron microscopy images

For each sample, a series of ten random 1282 × 957 nm fields containing gold-positive material was photographed on the JEOL JEM-1400 transmission electron microscope. Each image was subjected to local background subtraction followed by the creation of separate threshold masks for the gold particles and the surrounding aggregates. The threshold settings were standardized across all samples to reduce selection bias. To quantify the surface area of each aggregate containing at least 1 gold particle, the Analyze Particles plugin in ImageJ^60^ was applied to an image stack containing both threshold masks to generate gold particle positive aggregate regions of interest. To quantify the number of gold particles per aggregate, the Analyze Particles plugin in ImageJ was again used with the same regions of interest but the (denser) gold intensity threshold. The analysis was entirely automated, without user input after image selection.

### Statistics

All data were analyzed in Prism 7.0 (GraphPad Software, La Jolla, CA). The Shapiro-Wilk normality test was used to determine whether the distribution of the Aβ surface area measurements and gold particle counts were normally distributed. As the data sets displayed non-normal distributions in more than one group, Kruskal-Wallis ANOVA with Dunn *post hoc* test was used to compare groups. Spearman r correlations were performed to assess the relationship between the Aβ surface area and the number of gold particles.

### Ammonium acetate size exclusion chromatography

To assess the size distribution of amyloid beta aggregates using ammonium acetate during size exclusion chromatography, cortical tissue was homogenized and centrifuged at 100,000× g RCF as described above. Each sample was injected onto a Superdex 200 10/300 column equilibrated with 50 mM ammonium acetate, pH 8.5 at 4 °C and fractions collected. Each sample was subsequently injected on a Superdex 200 10/300 column equilibrated with 1x PBS at 4 °C and fractions collected. The fractions were assessed using the Aβ dimer equivalents ELISA.

### Liquid chromatography tandem mass spectrometry

Soluble Aβ aggregates were eluted from IP with 100% formic acid and dried under vacuum. The samples were then precipitated with the 2D Clean-Up Kit (GE Healthcare, Piscataway, NJ) to remove residual lipids, salt and polymers leached off of the beads used for immunoprecipitation during elution. The precipitated protein was resuspended in 100% formic acid. Samples were then subjected to C_8_ cleanup (Glygen) to separate larger proteins from Aβ peptides. C_8_ eluant was dried to completeness under vacuum and stored at −80 **°**C until analysis. Samples were resuspended in 1%/10%/5% formic acid/acetonitrile/methanol (v/v/v). The samples were analyzed by nanoLC-MS/MS on an Orbitrap Fusion mass spectrometer (ThermoFisher) in positive ion mode. Chromatographic separation was performed on an ACQUITY UPLC HSS T3 (360 μm OD × 75 μm ID) column packed with 10 cm C18 (1.8 μm, 100 Å, Waters) at 300 nL/min and heated to 60 °C. MS files (.raw) were imported into PEAKS (version 7.5, Waters) and searched against a UniprotKB/SwissProt Human database of canonical sequences (October 2015; 20,204 entries) appended with the cRAP contaminant database (December 2015 version, The Global Proteome Machine, www.thegpm.org/cRAP/index.html).

## Additional Information

**How to cite this article**: Esparza, T. J. *et al*. Soluble Amyloid-beta Aggregates from Human Alzheimer’s Disease Brains. *Sci. Rep.* **6**, 38187; doi: 10.1038/srep38187 (2016).

**Publisher's note:** Springer Nature remains neutral with regard to jurisdictional claims in published maps and institutional affiliations.

## Supplementary Material

Supplementary Information
