# Supplementary material for: Soluble Amyloid-beta Aggregates from Human Alzheimer’s Disease Brains
Source: Sci Rep. 2016 Dec 5;6:38187. doi: 10.1038/srep38187 (PMC5137165; doi:10.1038/srep38187)
Supplement: Supplementary Information [file srep38187-s1.pdf]

Soluble Amyloid-beta Aggregates from Human Alzheimer's Disease Brains

Thomas J. Esparza, Norelle C. Wildburger, Hao Jiang, Mihika Gangolli, Nigel J. Cairns, Randall J. Bateman and David L. Brody

SUPPLEMENTARY INFORMATION:

SUPPLEMENTARY FIGURES 2

Supplementary Fig. 1: Representative ELISA standard curves for 96 well plate format 2
Supplementary Fig. 2: The impact of detergent and serial extractions 3
Supplementary Fig. 3: CHAPS does not induce aggregation of synthetic Aβ_{1-42} 4
Supplementary Fig. 4: Assessing common detergents for effects on aggregation 4
Supplementary Fig. 5: Effect of blocking with 1% BSA on Aβ loss during incubation 5
Supplementary Fig. 6: Effect of blocking with 0.05% BSA on size exclusion chromatography 5
Supplementary Fig. 7: Separation of soluble Aβ aggregates using a centrifugal concentrator 6
Supplementary Fig. 8: 35% Ammonium sulfate precipitation of soluble Aβ aggregates 7
Supplementary Fig. 9: Alternative chromatographic methods 8
Supplementary Fig. 10: Assessment of immunoprecipitation elution buffers. 9
Supplementary Fig. 11: Aβ immunohistochemistry 10
Supplementary Fig. 12: Additional immunoelectron microscopic examples 11
Supplementary Fig. 13: Correlation between the surface area and number of immuno-gold labels for the soluble Aβ aggregates 12
Supplementary Fig. 14: Correlation between the surface area and number of immuno-gold labels for the 100,000 x g pellet, insoluble Aβ aggregates 13
Supplementary Fig. 15: Additional mass spectrometry data for Aβ_{1-40} and Aβ_{1-42} 14
Supplementary Fig. 16: Soluble Aβ aggregates from human brain assessed by SEC using PBS vs. 50 mM ammonium acetate as the mobile phase 15

SUPPLEMENTARY TABLES 16

Supplementary Table 1: Yield of soluble Aβ aggregates in 6 human AD brain preparations 16
Supplementary Table 2: Previous reports of soluble Aβ aggregates from human brain 17

SUPPLEMENTARY DISCUSSION 21

SUPPLEMENTARY REFERENCES 23

SUPPLEMENTARY FIGURES

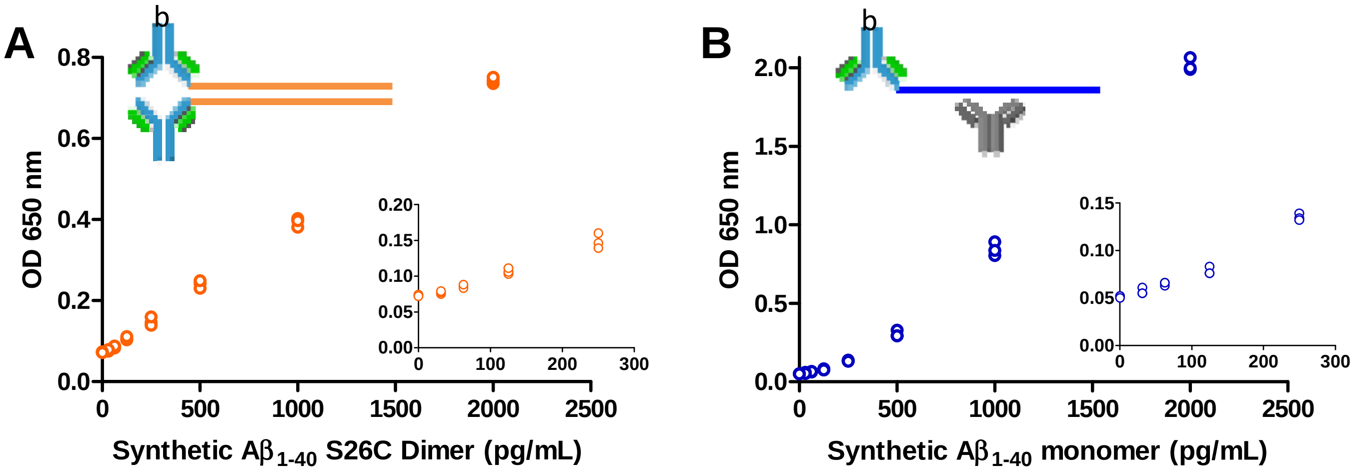

Supplementary Fig. 1: Representative ELISA standard curves for 96 well plate format. **A.** Soluble Aβ aggregate assay. Monoclonal antibody HJ3.4 specific to the N-terminus of Aβ as the capture antibody, and the same (biotinylated) HJ3.4 as the detection antibody. Standard curve made from synthetic $A\beta_{1\text{-}40\ Ser26Cys}$ dimer[1]. *Insets:* low concentration range, demonstrating lower limits of sensitivity. **B.** $A\beta_{1\text{-}x}$ assay. Monoclonal antibody HJ5.1 specific to the mid-domain of Aβ as the capture antibody and biotinylated HJ3.4 as the detection antibody. Linearity, sensitivity to 31.25 pg/mL, and low variability between replicates demonstrated for both assays.

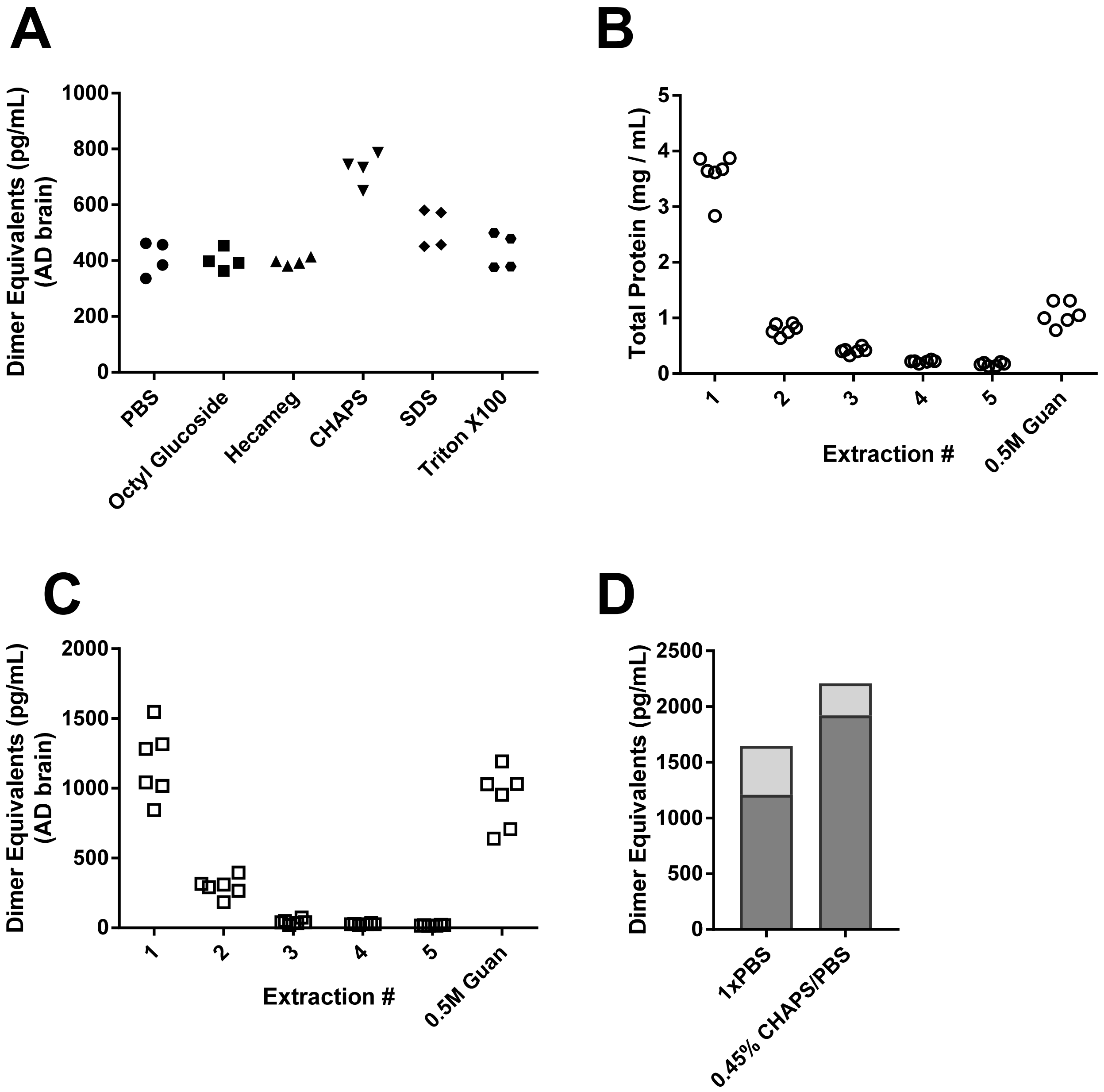

Supplementary Fig. 2: The impact of detergent and serial extractions on yields of soluble Aβ aggregates from human Alzheimer's disease cortical tissue. **A.** Comparison of sub-critical micelle concentration of detergents on yields of soluble Aβ aggregate yield during homogenization. **B.** Serial extraction of total protein measured by BCA assay from CDR3 cortical tissue with PBS + 0.45% CHAPS. Even after 5 extractions, 0.5M guanidine extraction yielded additional protein. **C.** Serial extraction of soluble Aβ aggregates from CDR3 cortical tissue with PBS + 0.45% CHAPS. Soluble Aβ aggregates were found primarily during the first and to a lesser extent the second extraction. Even after 5 extractions, further extraction using 0.5M Guanidine, a chaotrope known to solubilize Aβ plaques, yielded additional Aβ aggregates. This result indicated that the serial extractions in PBS + 0.45% CHAPS extracted a finite pool of Aβ but left a relatively insoluble pool of Aβ behind which could still be extracted with Guandine. **D.** PBS+0.45% CHAPS extraction yielded more soluble Aβ aggregates after initial homogenization (dark bars) and two-step homogenization (light bars) than PBS without CHAPS.

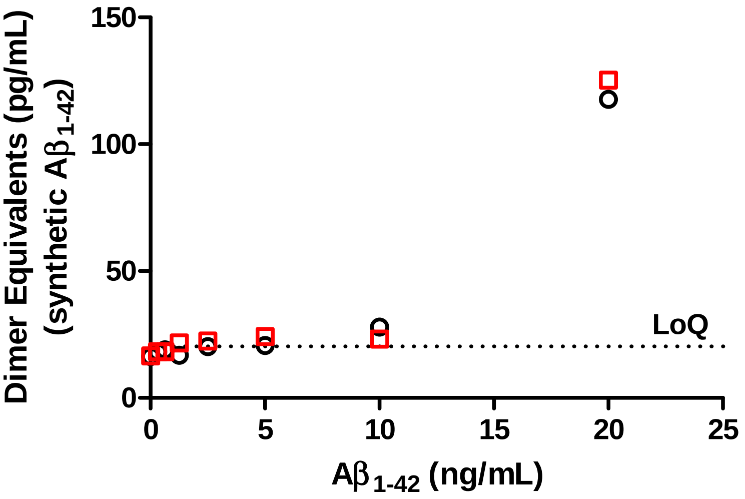

Supplementary Fig. 3: Sub-critical micelle concentration of CHAPS in PBS does not induce aggregation of synthetic A$\beta_{1\text{-}42}$. 0.45% CHAPS in PBS (red squares) during homogenization of cognitively normal cortical tissue spiked with an increasing amount of synthetic A$\beta_{1\text{-}42}$ does not induce more aggregation signal than a matched PBS homogenate (black circles).

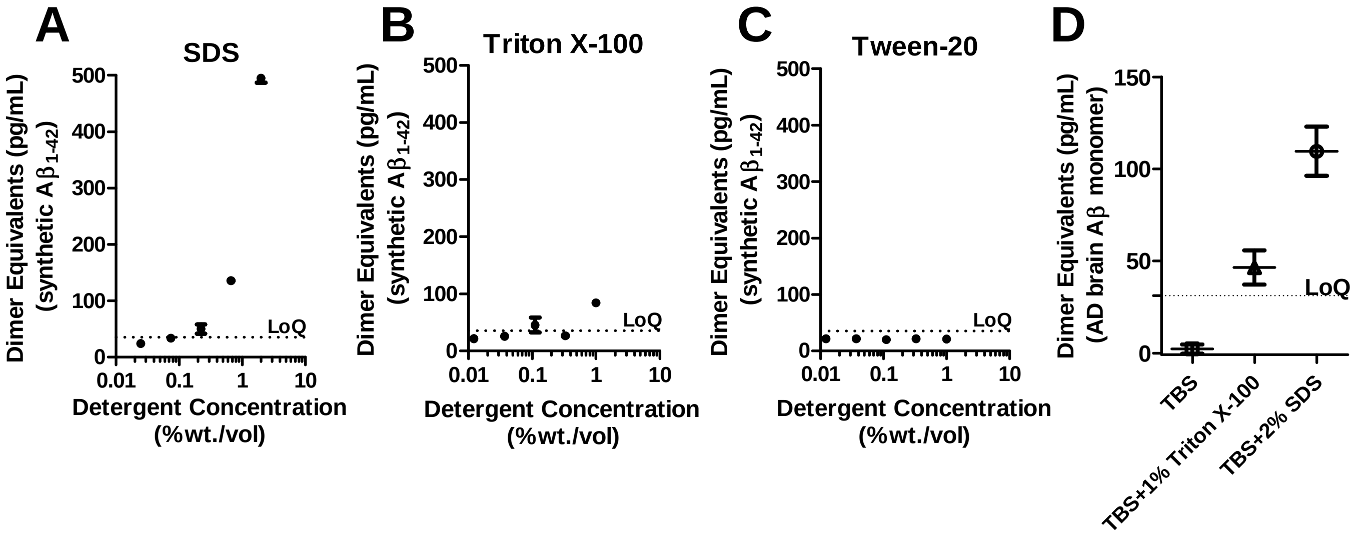

Supplementary Fig. 4: Assessing common detergents for effects on aggregation of monomeric Aβ. A-C. Titration of concentrations for (**A**) SDS, (**B**) Triton™ X-100, and (**C**) Tween® 20, was used to homogenize cognitively normal cortical tissue spiked with 2 ng/mL of synthetic A$\beta_{1\text{-}42}$. The resulting homogenates were immediately centrifuged at 100,000 x g RCF and assessed by ELISA for soluble Aβ aggregates. **D.** Soluble AD-brain derived Aβ monomers at 2 ng/mL were spiked into cognitively normal human cortical control tissue and homogenized in TBS, TBS+1% Triton X-100, or TBS+ 2% SDS. *Ex vivo* aggregation of Aβ monomers was observed with 1% Triton X-100 and 2% SDS detergents.

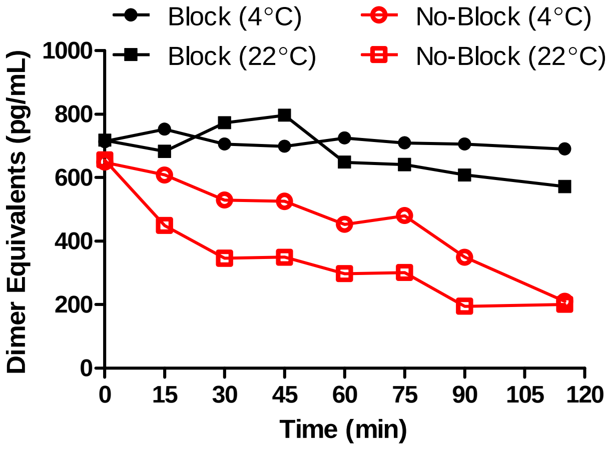

Supplementary Fig. 5: Effect of blocking with 1% bovine serum albumin (BSA) on Aβ loss during incubation. Microcentrifuge tubes blocked with 1% BSA demonstrate a minimal loss of Aβ aggregate in a 100,000 x g RCF clarified cortical homogenate on ice or at room temperature. Conversely, unblocked tubes display a rapid loss of Aβ during incubation with moderate temperature dependence.

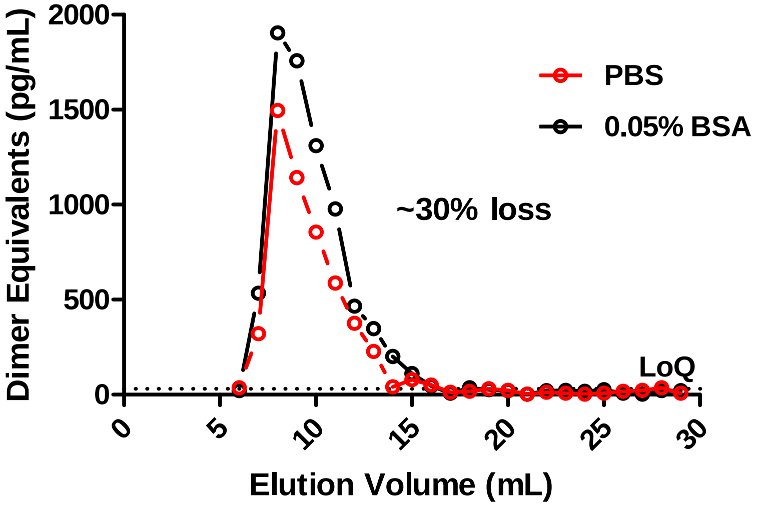

Supplementary Fig. 6: Effect of blocking with 0.05% BSA on size exclusion chromatography. Approximately 30% of the injected soluble Aβ aggregates from human AD brain lysate were lost during a single pass over the Superdex 200 10/300 column as demonstrated by the PBS only sample. This loss was greatly reduced with the addition of 0.05% BSA to the mobile phase buffer. No changes in the size distribution were observed. With SEC including bovine serum albumin in the mobile phase, the recovery of soluble Aβ aggregates was 86%. The recovery was calculated by measuring the concentration of soluble Aβ aggregates in the initial lysates and comparing it with the concentrations in each of the fractions.

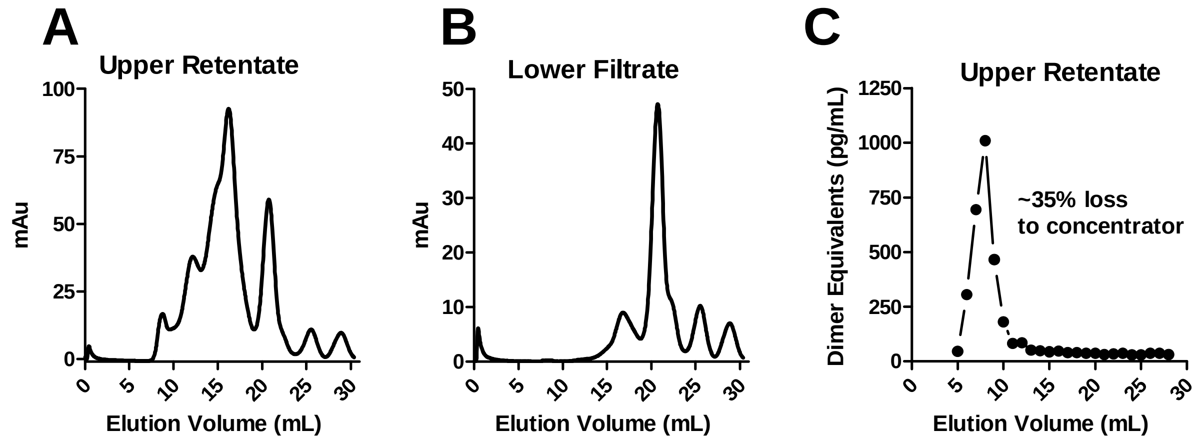

Supplementary Fig. 7: Separation of soluble Aβ aggregates from monomers by using a molecular weight cut off centrifugal concentrator. Using a 100 kDa MWCO concentrator, a freshly prepared cortical homogenate was spun at 100,000 x g and then centrifuged in the concentrator until 10-fold concentrated. **A.** Size exclusion chromatography of the retentate demonstrated both high and low molecular weight material. **B.** Size exclusion chromatography of the filtrate demonstrated exclusively low molecular weight material. **C.** Soluble Aβ aggregates were found in the retentate without apparent change in size, but with loss >30% during concentration.

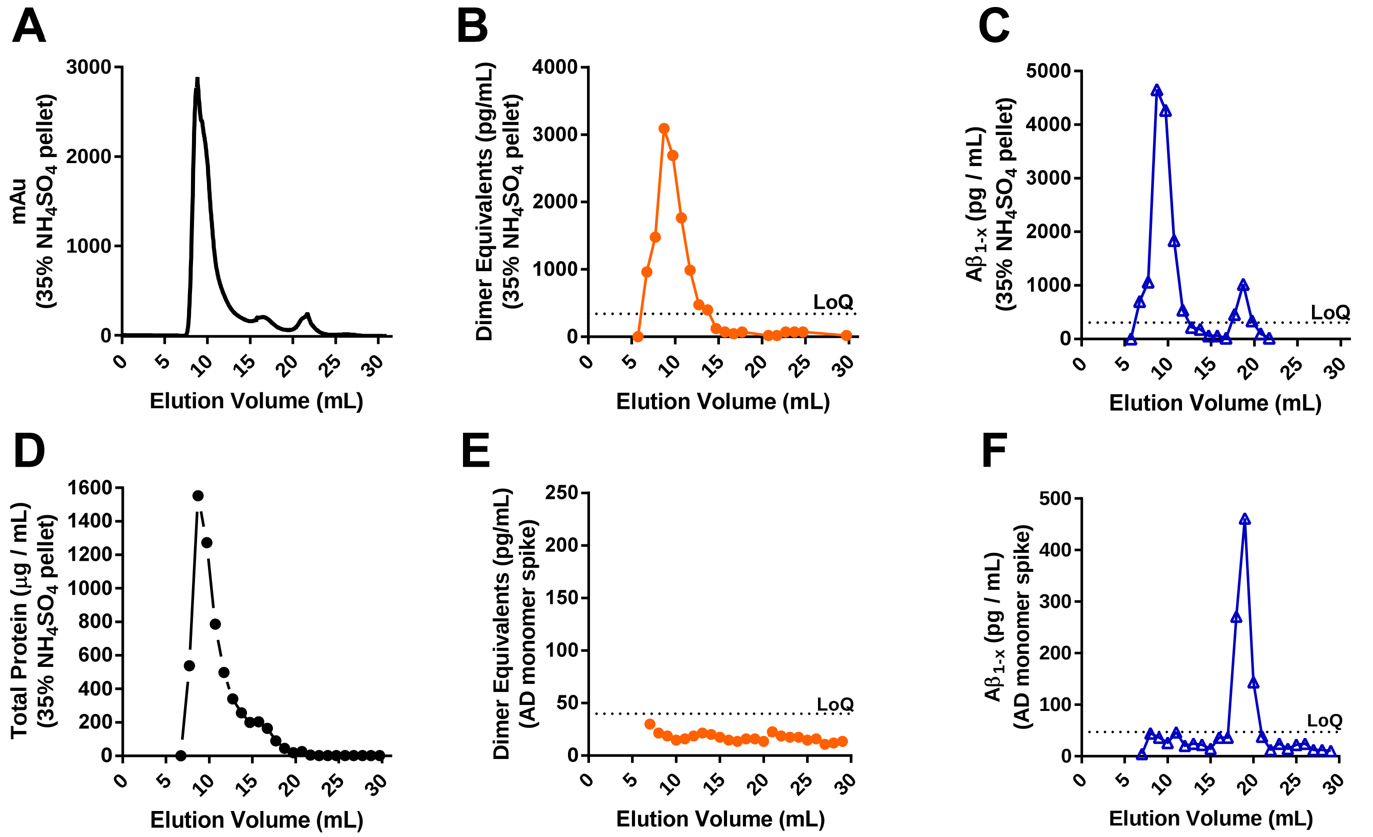

Supplementary Fig. 8: 35% Ammonium sulfate precipitation of soluble Aβ aggregates as a method of enrichment. Following precipitation and resuspension, the sample was run over the Superdex 200 column. **A.** Total protein assessed by BCA assay indicated largely high molecular weight species. **B.** The soluble Aβ aggregate ELISA indicated no apparent effect on the size forms of the aggregates. **C.** The $A\beta_{1-x}$ ELISA indicates presence of both high molecular weight and low molecular weight (likely monomeric) Aβ in the precipitates. **D-F.** Cognitively normal cortical tissue spiked during homogenization with 2 ng/mL AD-derived Aβ monomer and precipitated by 35% ammonium sulfate does not induce *ex vivo* aggregation.

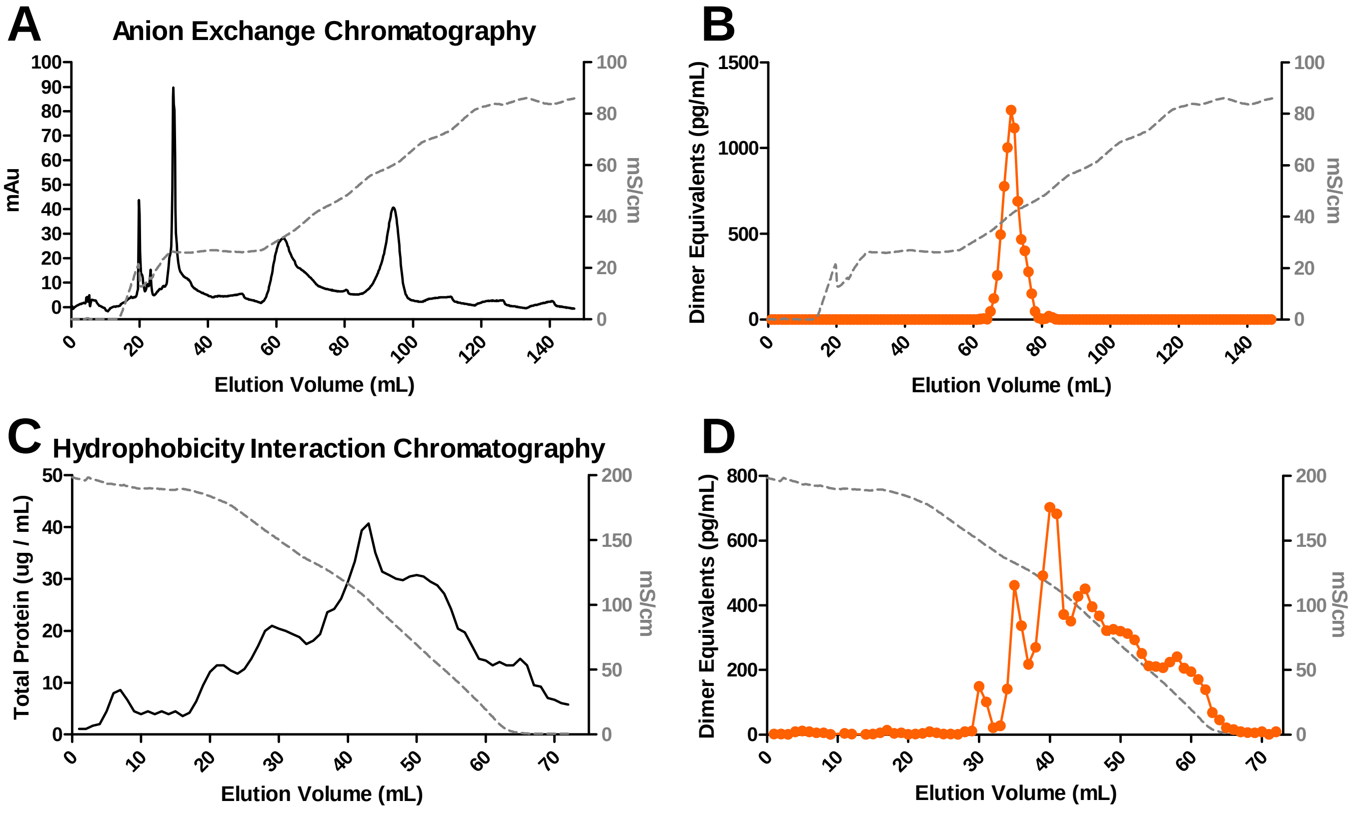

Supplementary Fig. 9: Alternative chromatographic methods provide some utility for enrichment and separation of soluble Aβ aggregates. A-B. Anion exchange chromatography using a HiScreen Q column bound soluble Aβ aggregates from cortical homogenates, and they eluted in a single discrete but wide peak with modest quantities of other protein. **C-D.** Hydrophobicity interaction chromatography using a HiScreen Butyl column bound soluble Aβ aggregates, and they eluted in a complex and heterogeneous fashion with substantial amounts of other protein. This could potentially provide a method to segregate soluble Aβ aggregates displaying differential hydrophobicity characteristics.

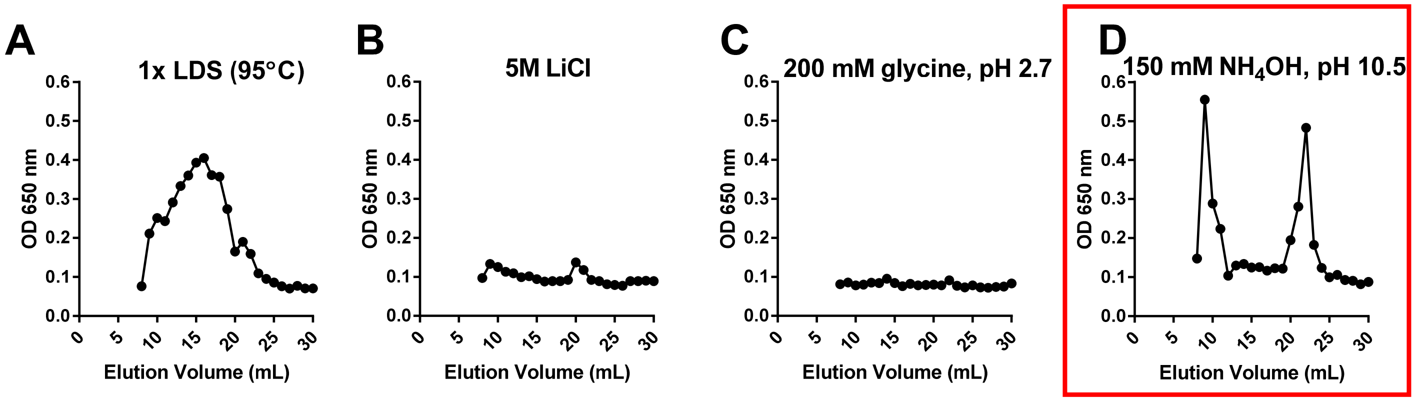

Supplementary Fig. 10: Assessment of immunoprecipitation elution buffers. A bulk immunoprecipitation of soluble Aβ from human CDR3 brain lysates overnight at 4°C with dual antibody HJ3.4 and HJ5.1 conjugated beads was performed, and the bead volume was split evenly between 4 elution conditions. The samples were eluted sequentially to allow immediate size exclusion chromatography analysis and assessment of Aβ size distribution. **A.** Elution in Lithium dodecyl sulfate (1xLDS) buffer at 95°C dramatically altered the size distribution of the soluble Aβ. **B.** Elution in 5M lithium chloride resulted in poor recovery of Aβ. **C.** Elution in 200 mM glycine, pH 2.7 resulted in almost no recovery of Aβ. **D.** Elution in 150 mM ammonium hydroxide, pH 10.5 resulted nearly complete recovery of Aβ without changing the size distribution, except for sharpening the peaks on size exclusion chromatography.

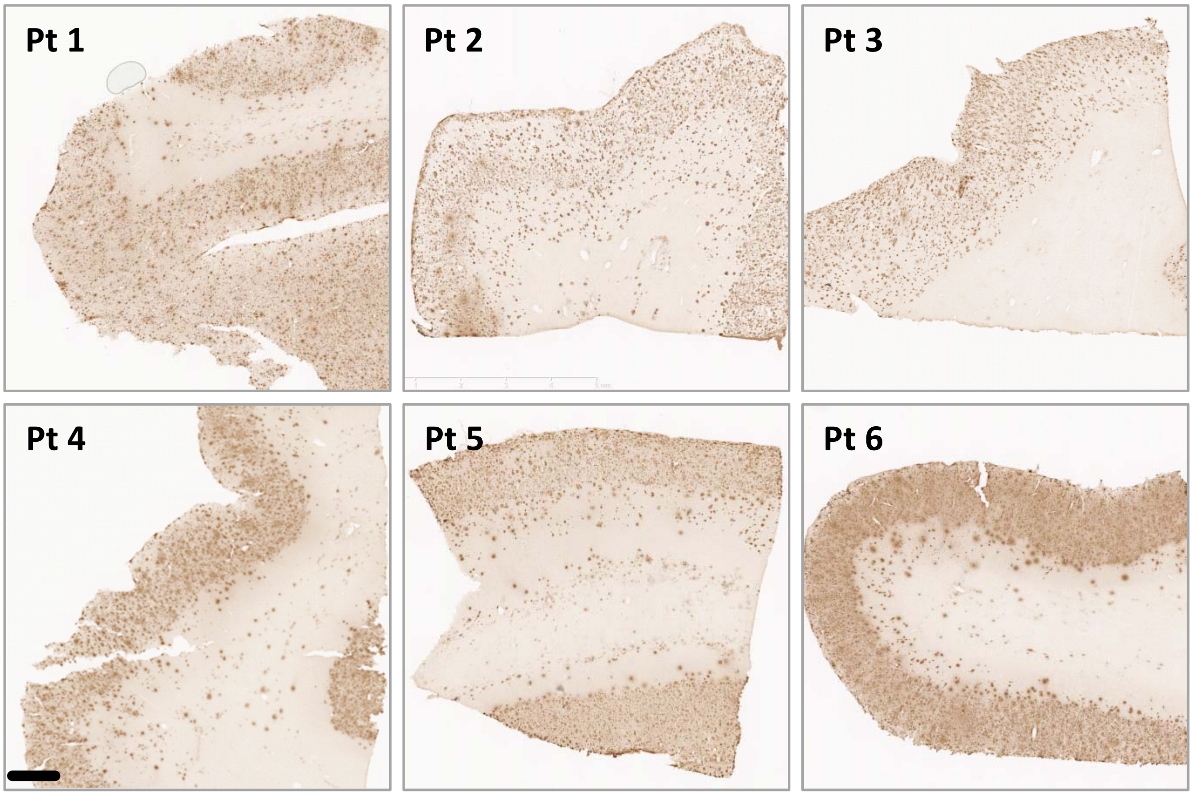

Supplementary Fig. 11: **Aβ immunohistochemistry on slices made from the frozen tissue blocks used for soluble Aβ aggregate preparations.** All donors were assessed at CDR3 (advanced dementia) at the time of death. Tissue was immunostained with the Aβ N-terminal specific mAb 82E1 (IBL International) at 1 µg/mL with methods that were otherwise identical to those used previously[1]. Scale bar = 1 mm.

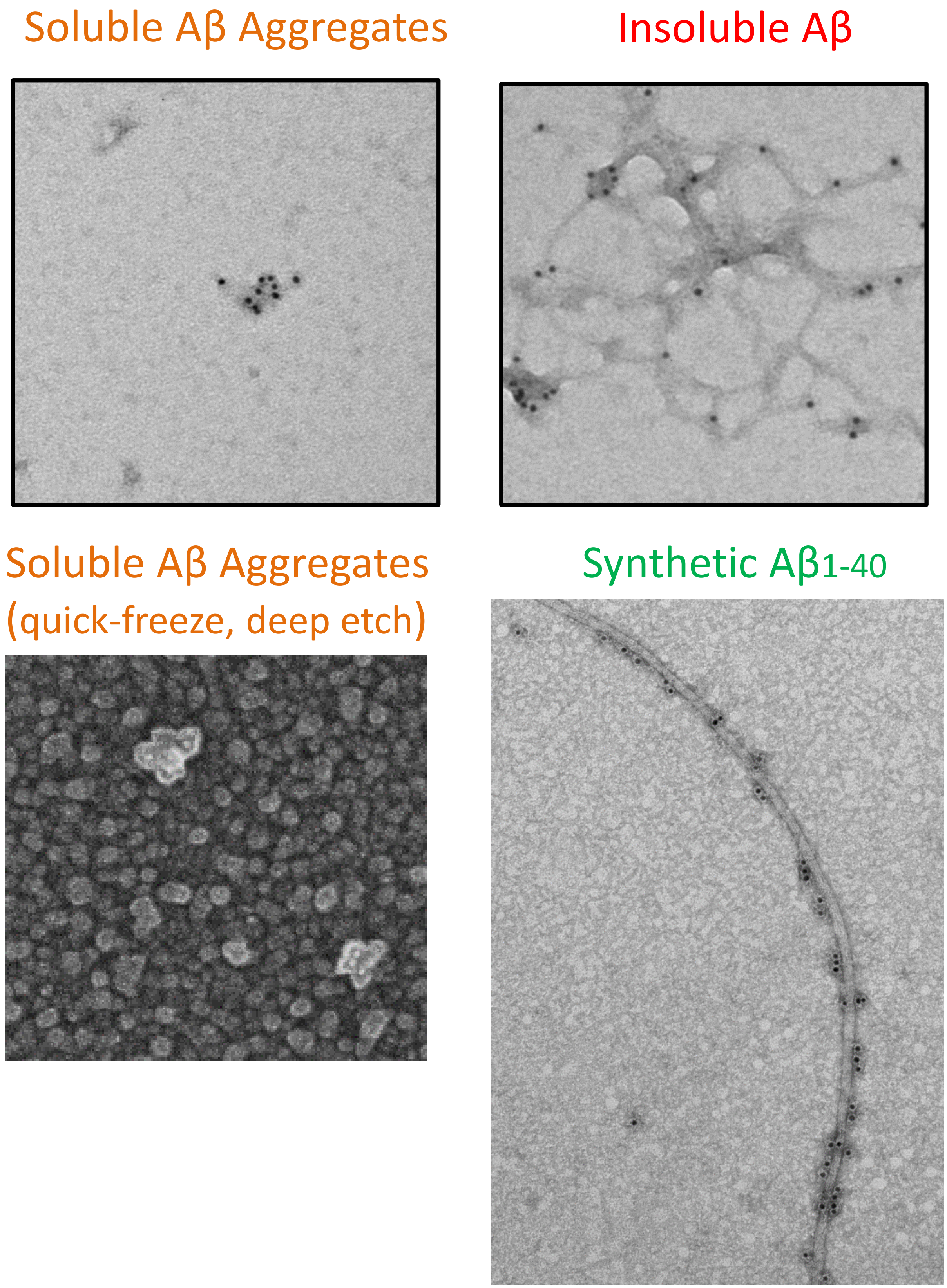

Supplementary Fig. 12: Additional immunoelectron microscopic examples of the morphological characteristics of soluble Aβ aggregates vs. insoluble aggregates from human AD brain vs. synthetic Aβ.

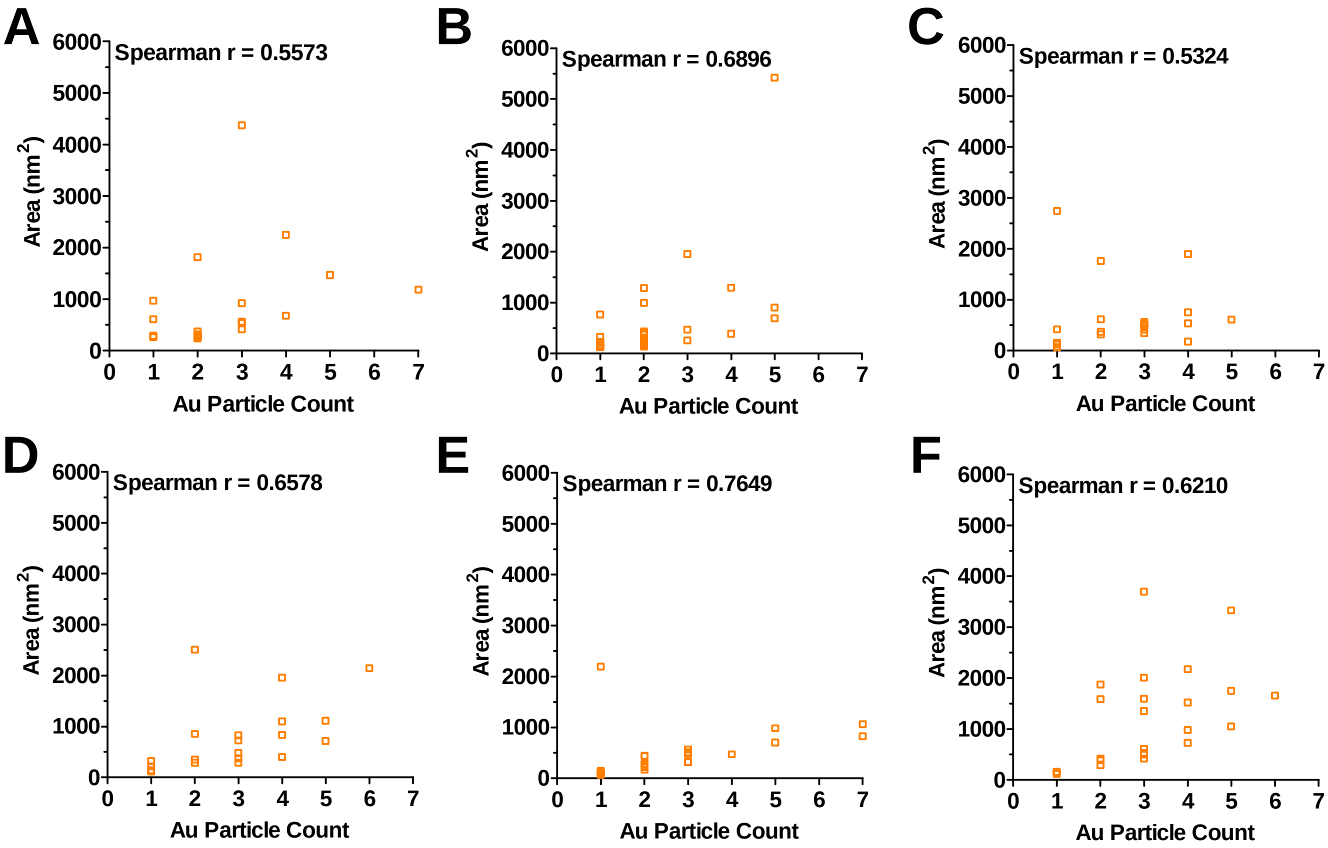

Supplementary Fig. 13: Correlation between the surface area and number of immuno-gold labels for the soluble Aβ aggregates. Data from patients 1-6 displayed in panels A-F. The Spearman correlations for each sample were statistically significant ($p<0.001$ to 0.007), though modest, indicating a heterogeneous relationship between aggregate size and number of N-terminal Aβ antibody binding sites.

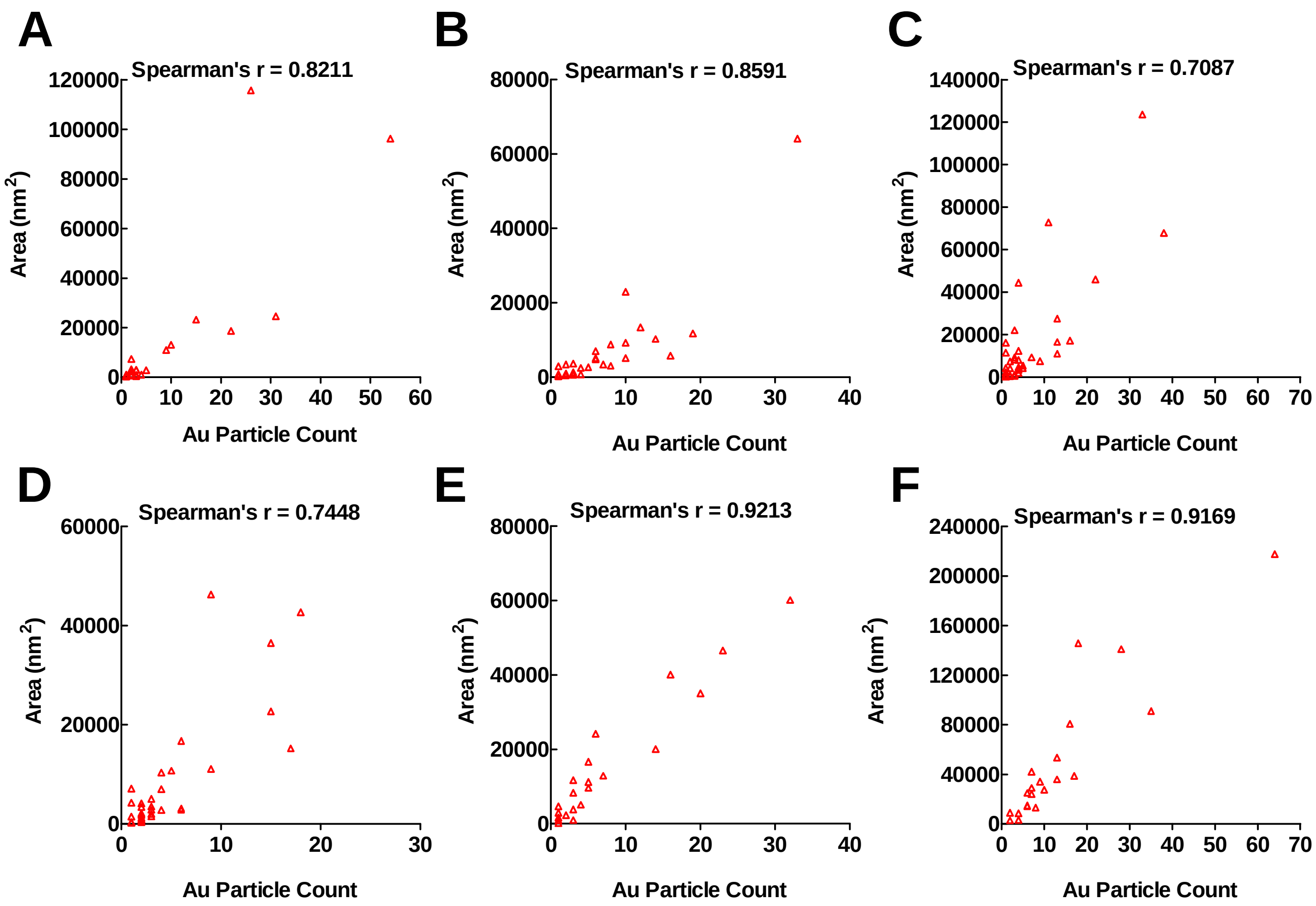

Supplementary Fig. 14: Correlation between the surface area and number of immuno-gold labels for the 100,000 x g pellet, insoluble Aβ aggregates. Data from patients 1-6 displayed in panels A-F. The Spearman correlations for each sample were highly significant (all $p<0.0001$). The correlations between area and number of immunogold labels for the insoluble Aβ aggregates were stronger than those between area and number of immunogold labels for the soluble Aβ aggregates (difference tests, $p<0.05$). The slopes of the correlations were between 1400 and 2400 nm^2 per gold particle.

A

#	b	b-H2O	b-NH3	b (2+)	Seq	y	y-H2O	y-NH3	y (2+)	#
1	116.0348	98.0242	99.0078	58.5174	D					40
2	187.0720	169.0970	170.0449	94.0359	A	4213.1289	4195.1182	4196.1016	2107.0645	39
3	316.1134	298.1039	299.1730	158.5572	E	4142.0918	4124.0811	4125.0645	2071.5459	38
4	463.1821	445.1723	446.1559	232.0914	F	4013.0488	3995.0383	3996.0217	2007.0244	37
5	619.2809	601.2734	602.2570	310.1420	R	3865.9805	3847.9700	3848.9534	1933.4902	36
6	756.3448	738.3323	739.3159	378.6714	H	3709.8794	3691.8689	3692.8523	1855.4397	35
7	871.3683	853.3593	854.3428	436.1849	D	3572.8206	3554.8101	3555.7935	1786.9103	34
8	958.4019	940.3913	941.3749	479.7009	S	3457.7937	3439.7832	3440.7666	1729.3969	33
9	1015.4233	997.4127	998.3963	508.2116	G	3370.7615	3352.7510	3353.7344	1685.8807	32
10	1178.4866	1160.4761	1161.4596	589.7433	Y	3313.7400	3295.7295	3296.7129	1657.3700	31
11	1307.5291	1289.5187	1290.5022	654.2646	E	3150.6768	3132.6663	3133.6497	1575.8384	30
12	1406.6008	1388.5872	1389.5707	703.8060	V	3021.6343	3003.6238	3004.6072	1511.3171	29
13	1543.6748	1525.6460	1526.6295	772.3282	H	2922.5657	2904.5552	2905.5386	1461.7828	28
14	1680.7058	1662.7050	1663.6885	840.8577	H	2785.5068	2767.4963	2768.4797	1393.2534	27
15	1808.7878	1790.7635	1791.7471	904.8870	Q	2648.4480	2630.4375	2631.4209	1324.7240	26
16	1936.8632	1918.8585	1919.8420	968.9345	K	2520.3894	2502.3789	2503.3623	1260.6947	25
17	2049.9531	2031.9365	2032.9261	1025.4835	L	2392.2944	2374.2839	2375.2673	1196.6472	24
18	2149.0164	2131.0110	2131.9944	1075.0107	V	2279.2104	2261.2000	2262.1833	1140.1052	23
19	2296.0977	2278.0793	2279.0627	1148.5449	F	2180.1418	2162.1313	2163.1147	1090.5709	22
20	2443.1665	2425.1477	2426.1311	1222.0791	F	2033.0735	2015.0630	2016.0465	1017.0367	21
21	2514.1814	2496.1848	2497.1682	1257.6235	A	1886.0051	1867.9946	1868.9781	943.5026	20
22	2643.2288	2625.2275	2626.2109	1322.1254	E	1814.9680	1796.9575	1797.9410	907.9840	19
23	2758.2668	2740.2544	2741.2378	1379.6405	D	1685.9254	1667.9149	1668.8984	843.4627	18
24	2857.3428	2839.3228	2840.3062	1429.1666	V	1570.8984	1552.8879	1553.8715	785.9492	17
25	2914.3691	2896.3442	2897.3276	1457.6918	G	1471.8301	1453.8196	1454.8031	736.4150	16
26	3001.4009	2983.3762	2984.3596	1501.2026	S	1414.8086	1396.7981	1397.7816	707.9043	15
27	3115.4297	3097.4192	3098.4026	1558.2148	N	1327.7766	1309.7661	1310.7496	664.3883	14
28	3243.5222	3225.5142	3226.4976	1622.2623	K	1213.7336	1195.7231	1196.7067	607.3668	13
29	3300.5532	3282.5356	3283.5190	1650.7731	G	1085.6387	1067.6282	1068.6117	543.3063	12
30	3371.5894	3353.5728	3354.5562	1686.3060	A	1028.6173	1010.6067	1011.5903	514.8087	11
31	3484.6729	3466.6707	3467.6401	1742.8336	I	957.5801	939.5696	940.5532	479.2901	10
32	3597.7524	3579.7410	3580.7244	1799.3757	I	844.4129	826.4855	827.4691	422.7480	9
33	3654.7725	3636.7522	3637.7458	1827.8704	G	731.4120	713.4014	714.3850	366.2060	8
34	3767.8557	3749.8501	3750.8298	1884.4285	L	674.3906	656.3800	657.3760	337.6953	7
35	3898.9058	3880.8870	3881.8704	1949.9487	M	561.3065	543.3063	544.2795	281.1532	6
36	3997.9658	3979.9553	3980.9387	1999.4829	V	430.2653	412.2546	413.2390	215.6330	5
37	4054.9873	4036.9768	4037.9602	2027.9937	G	331.1968	313.1872	314.1872	166.0988	4
38	4112.0088	4093.9983	4094.9817	2056.5044	G	274.1763	256.1656	257.1313	137.5881	3
39	4211.0771	4193.0664	4194.0503	2106.0386	V	217.1549	199.1804	200.1277	109.0773	2
40					V	118.0863	100.0757	101.0592	59.5431	1

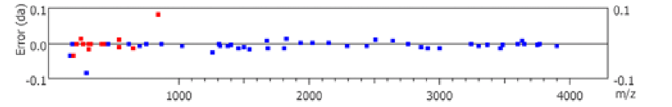

B

#	b	b-H2O	b-NH3	b (2+)	Seq	y	y-H2O	y-NH3	y (2+)	#
1	116.0348	98.0242	99.0078	58.5174	D					42
2	187.0711	169.0968	170.0449	94.0359	A	4397.2500	4379.2393	4380.2227	2199.1250	41
3	316.1132	298.1039	299.1727	158.5572	E	4326.2129	4308.2021	4309.1855	2163.6064	40
4	463.1835	445.1723	446.1559	232.0914	F	4197.1704	4179.1597	4180.1431	2099.0852	39
5	619.2775	601.2734	602.2570	310.1420	R	4050.1018	4032.0913	4033.0747	2025.5509	38
6	756.3563	738.3323	739.3159	378.6714	H	3894.0005	3875.9900	3876.9734	1947.5002	37
7	871.3708	853.3593	854.3428	436.1849	D	3756.9417	3738.9312	3739.8628	1878.9708	36
8	958.4019	940.3913	941.3749	479.7009	S	3641.9148	3623.9043	3624.8877	1821.4574	35
9	1015.4233	997.4127	998.3963	508.2106	G	3554.8828	3536.8723	3537.8557	1777.9414	34
10	1178.4866	1160.4761	1161.4596	589.7459	Y	3497.8613	3479.8508	3480.8342	1749.4307	33
11	1307.5316	1289.5187	1290.5022	654.2646	E	3334.7979	3316.7874	3317.7708	1667.8989	32
12	1406.5972	1388.5872	1389.5707	703.7988	V	3205.7554	3187.7449	3188.7283	1603.3777	31
13	1543.6564	1525.6460	1526.6295	772.3282	H	3106.6870	3088.6765	3089.6599	1553.8435	30
14	1680.7344	1662.7050	1663.6885	840.8577	H	2969.6279	2951.6174	2952.6008	1485.3140	29
15	1808.7740	1790.7635	1791.7471	904.8999	Q	2832.5691	2814.5586	2815.5420	1416.7845	28
16	1936.8690	1918.8585	1919.8420	968.9466	K	2704.5105	2686.5000	2687.4834	1352.7552	27
17	2049.9612	2031.9568	2032.9261	1025.4766	L	2576.4155	2558.4050	2559.3884	1288.7078	26
18	2149.0212	2131.0110	2131.9944	1075.0145	V	2463.3315	2445.3210	2446.3044	1232.1658	25
19	2296.0896	2278.0793	2279.0627	1148.5449	F	2364.2632	2346.2527	2347.2361	1182.6316	24
20	2443.1648	2425.1477	2426.1311	1222.0966	F	2217.1946	2199.1841	2200.1675	1109.0973	23
21	2514.1741	2496.1848	2497.1682	1257.5977	A	2070.1262	2052.1157	2053.0991	1035.5631	22
22	2643.2246	2625.2275	2626.2109	1322.1158	E	1999.0892	1981.0787	1982.0623	1000.0446	21
23	2758.2686	2740.2544	2741.2378	1379.6514	D	1870.0466	1852.0361	1853.0197	935.5233	20
24	2857.3408	2839.3228	2840.3062	1429.1666	V	1755.0197	1737.0092	1737.9927	878.0098	19
25	2914.3677	2896.3442	2897.3276	1457.7064	G	1655.9513	1637.9408	1638.9243	828.4756	18
26	3001.3831	2983.3762	2984.3596	1501.1934	S	1598.9298	1580.9193	1581.9028	799.9649	17
27	3115.4358	3097.4192	3098.4026	1558.2272	N	1511.8977	1493.8872	1494.8707	756.3563	16
28	3243.5283	3225.5142	3226.4976	1622.2623	K	1397.8549	1379.8444	1380.8279	699.4274	15
29	3300.5723	3282.5356	3283.5190	1650.7731	G	1269.7599	1251.7494	1252.7329	635.3799	14
30	3371.5879	3353.5728	3354.5562	1686.2916	A	1212.7384	1194.7279	1195.7114	606.8692	13
31	3484.6680	3466.6567	3467.6401	1742.8336	I	1141.7013	1123.6908	1124.6743	571.3212	12
32	3597.7607	3579.7410	3580.7244	1799.3757	I	1028.6173	1010.5991	1011.5903	514.8087	11
33	3654.7756	3636.7625	3637.7593	1827.8865	G	915.5332	897.5308	898.5062	458.2424	10
34	3767.8579	3749.8396	3750.8298	1884.4285	L	858.5117	840.5012	841.4847	429.7559	9
35	3898.9036	3880.8870	3881.8704	1949.9487	M	745.4277	727.4095	728.4007	373.2264	8
36	3997.9658	3979.9553	3980.9387	1999.4829	V	614.3315	596.3145	597.3713	307.6936	7
37	4054.9873	4036.9768	4037.9602	2027.9937	G	515.2560	497.3082	498.2917	258.1594	6
38	4112.0088	4093.9983	4094.9817	2056.5044	G	458.2424	440.2867	441.2703	229.6486	5
39	4211.0771	4193.0664	4194.0503	2106.0386	V	401.2225	383.2653	384.2652	201.1379	4
40	4310.1455	4292.1348	4293.1187	2155.5728	V	302.1531	284.1972	285.1925	151.6037	3
41	4423.2295	4405.2188	4406.2026	2212.1147	I	203.1389	185.1650	186.1237	102.0695	2
42					A	90.0549	72.0444	73.0279	45.5275	1

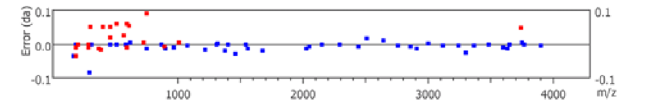

C

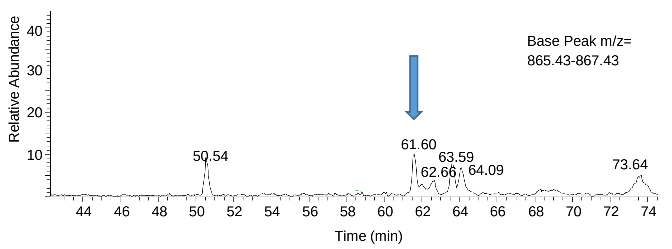

D

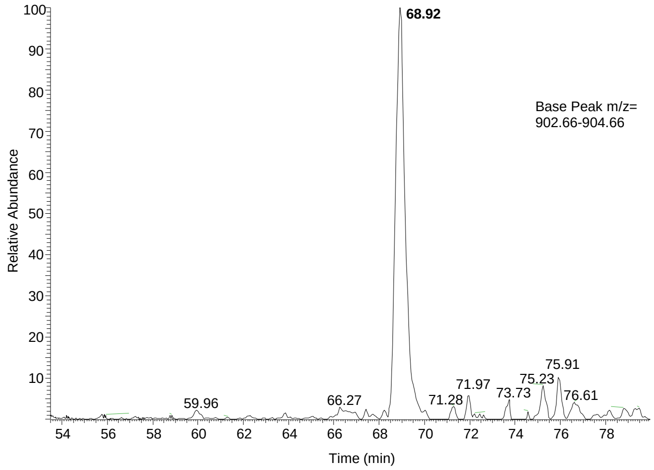

Supplementary Fig. 15: Additional mass spectrometry data for Aβ$_{1-40}$ and Aβ$_{1-42}$ detected in soluble Aβ aggregates from human brain. A. The *b*-ion and *y*-ion table for Aβ$_{1-40}$. Blue text indicates detected *b*-ions, red text indicated detected *y*-ions. Bottom graph indicates mass error relative to expected mass as a function of expected mass. **B.** The *b*-ion and *y*-ion table for Aβ$_{1-42}$. **C.** Extracted ion chromatogram for Aβ$_{1-40}$, indicating the time in minutes when the full-length Aβ$_{1-40}$ came off the C$_{18}$ chromatographic column. The material eluting at the peak at 61.60 minutes (blue arrow) was used for analyses. Relative abundance based on peak area was 1.62 x10^{7}. **D.** Extracted ion chromatogram for Aβ$_{1-42}$ with a single major peak at 68.92 minutes. Relative abundance based on peak area was 6.17 x10^{7}.

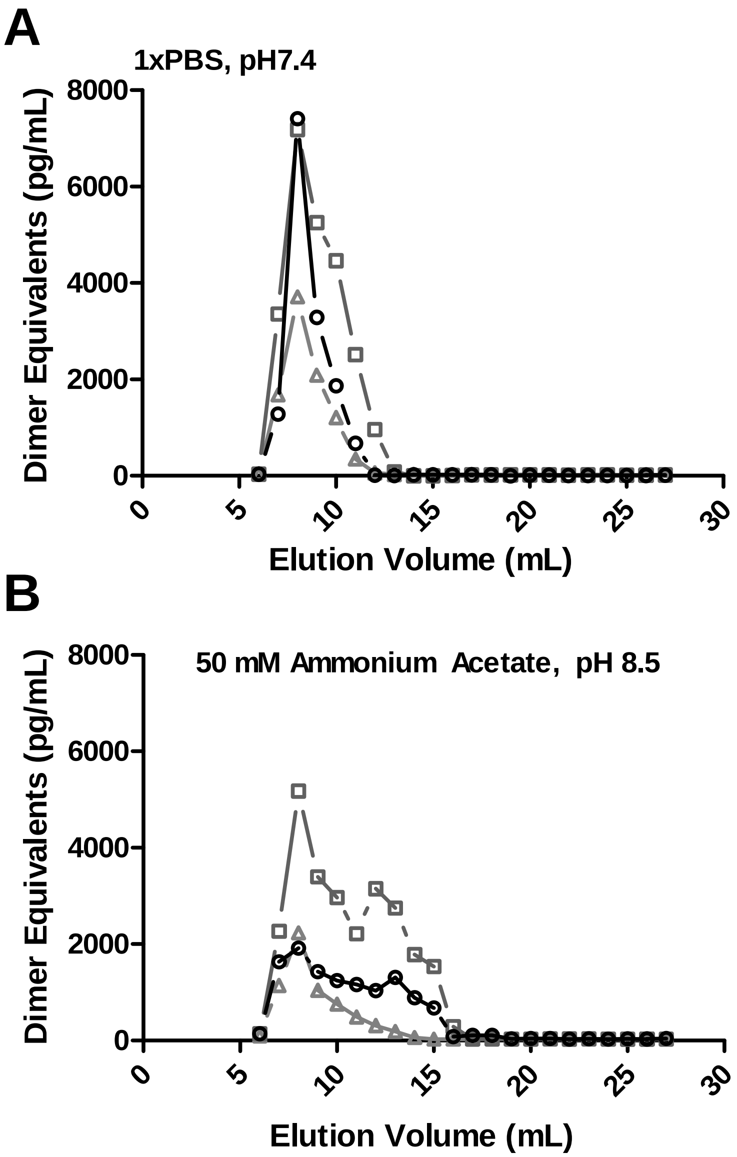

Supplementary Fig. 16: Soluble Aβ aggregates from human brain assessed by SEC using PBS vs. 50 mM ammonium acetate as the mobile phase. Fresh cortical homogenates from 3 different brain samples were prepared in 1xPBS and stored at -80°C for analysis. **A.** Aliquots from the homogenates were run on the Superdex 200 column using 1xPBS as the mobile phase. Each fraction was assessed using the soluble Aβ aggregate ELISA. **B.** Aliquots from the same 3 homogenates were run on the same Superdex 200 column using 50 mM ammonium acetate, pH 8.5 as the mobile phase. Each fraction was assessed using the same soluble Aβ aggregate ELISA. The results indicate that apparent dissociation occurs in the presence of ammonium acetate, but the extent varies from sample to sample.

SUPPLEMENTARY TABLES

Supplementary Table 1: Yield of soluble Aβ aggregates in 6 human AD brain preparations

Sample #	Region	Tissue mass (g)	Total Protein Eluted (ng, Nano-Orange)	Soluble Aβ Aggregate Eluted (ng dimer equivalents)	Yield (Soluble Aβ aggregates as % of total protein)
1	Frontal Ctx	2.41	2972.82	9.73	0.327
2	Frontal Ctx	1.96	4503.63	7.26	0.161
3	Frontal Ctx	2.04	2155.43	12.95	0.601
4	Frontal Ctx	2.27	5446.35	6.35	0.117
5	Frontal Ctx	2.13	2384.21	8.49	0.356
6	Frontal Ctx	2.32	2887.19	5.39	0.187

Supplementary Table 2: Previous reports of soluble Aβ aggregates from human brain

Reference	Preparation	Size forms	Structure	toxicity	*Ex vivo* aggregation and specificity controls?
Roher et al. 1991[2]	SDS, sucrose gradient, Formic acid lysates	Mono dimer trimer by SEC	No data (thioflavin positive)	Killed sympathetic neurons in culture	No (probably derived from plaques)
Frackowiak et al. 1994[3]	cerebral blood vessels in TBS +0.65% NP40 detergent	4.5, 9, 13.5, 17.5 kDa by SDS-PAGE WB	No data	No data	No (detergent effects possible, 4G8 6E10 APP fragments possible)
Kuo et al. 1996[4]	TBS lysate, 135k x g sup., 220k x g sup.	<10 kDa, 10-30 kDa, 30-100 kDa, >100kDa by differential filtration	No data	No data	No (not clear if oligomer, APP or Aβ bound to other molecules)
Roher et al. 1996[5]	Sucrose, formic acid, SDS lysate, (plaque derived) Superose12 SEC	9, 13.5 kDa on SDS-PAGE WB	CNBr cleavage AFM Ellipsoids 3-4 nm diameter	Cultured neuron killing only with microglia present	No. (SDS effects possible)
McLean et al. 1999[6]	PBS lysate, 175k x g sup., freeze dried, SDS 8M urea	>8 kDa, >12 kDa on SDS-PAGE WB	No data	No data	No. (SDS effects possible) (not clear if APP or Aβ bound to other molecules)
Gong et al. 2003[7]	F12 media lysate, 100k x g sup., dot blot	56 kDa, pI 5.6 on 2 dimensional gel	Antibodies selective for synthetic oligomers	No data (binding to cultured neurons)	No. (SDS effects possible)
Shankar et al. 2008[8]	TBS lysate, 175kG sup., SEC in ammonium acetate.	Large (>60 kDa) and 8-16 kDa ~dimer on LDS-PAGE WB	Mass spec on guanidine extracts: (not in TBS soluble) amino acid 17-28 & 29-40. Weak N-terminal antibody binding.	Slice LTP impaired & LTD enhanced by TBS extracts. Passive avoidance impaired by icv infusion in rats. Spine loss in culture after lyophilization.	No. (Ammonium Acetate, LDS, lyophilzation. effects possible) But toxicity in original lysates
Noguchi et al. 2009[9]	TBS lysate, 104k x g sup.	>100 kDa by filtration	10-15 nm spheroids on TEM, A11 neg., MALDI identification of $A\beta_{1-40}$ & $A\beta_{1-42}$	Apoptosis of primary rat septal neurons, Degeneration of hMSC-derived neuron-like cells.	Yes. Spiked synthetic $A\beta_{1-42}$ into control brains and found no aggregation by TEM.
Mc Donald et al.	TBS lysate, 91k x g	7.5 kDa	3D6 positive (Aβ	Differences	No.

2010[10]	sup., anti-Aβ IP.	'dimer'<br>12.1 kDa 'trimer' on SDS PAGE WB	N-terminal)<br>$A\beta_{x-40} > A\beta_{x-42}$	between demented and nondemented, but some overlap	(SDS effects possible)
Barry et al. 2011[11]	TBS lysate, 91k x g sup., exchange into ammonium acetate	7.5 kDa on SDS PAGE WB	No data	inhibited *in vivo* LTP in rat	No.<br>(Ammonium acetate effects possible)
Freir et al. 2011[12]	TBS lysate, 91k x g sup., dialysis into TBS x 48h at 4C	7.5 kDa on SDS PAGE WB	No data	Mouse brain slice LTP inhibited.<br>Rat *in vivo* LTP inhibited<br>Blocked by anti PrP antibody	No, but minimal manipulation for toxicity experiments (SDS effects possible for WB)
Lasanga-Reeves et al. 2011[13]	PBS lysate 78k x g sup., IP with novel conformational antibody	>100 kDa smear on SDS PAGE WB	11-14 nm diameter with 2.5-4 nm "pores" by TEM	No data	No<br>(SDS effects possible)<br>(No immunoEM confirmation of Aβ)
Zou et al. 2011[14]	TBS+0.5% Nonidet, 0.5% deoxycholate lysate, 100k x g sup., Superdex 200 in 2% sarcosyl	High MW near 669 kDa, detected as smaller forms on SDS PAGE 6E10 WB	Includes aggregated PrP, confirmed by co-IP mainly in insoluble.<br>Co-IP with g5p	No data	No.<br>(Detergent effects possible, APP antibody binding possible)
Jin et al. 2011[15]	TBS lysate, 175k x g sup., IP, LDS elution, SEC in ammonium acetate	~7 kDa 'dimer' on SDS-PAGE WB	No data	Tau positive & neuritic beading in mature cultured neurons at 0.5 nM x 3 d	No<br>(ammon acetate, LDS, & lyophilization effects possible)
Bjorklund et al. 2012[16]	Synaptic fractionation of hippocampus by sucrose gradient 100k x g sup, pH6 1M tris, 1% triton, 1% SDS.	8, 12, 16 kDa on SDS-PAGE 6E10 & NU4 WB from postsynaptic fractions	No data	No data (aggregates detected in tissue from demented but not non-demented subjects)	No.<br>(Detergents effects possible, APP fragments possible)
Mc Donald et al. 2012[17]	TBS lysate, 220k x g sup. then 1% triton then 88% FA	7 kDa & 18 kDa on SDS PAGE WB	No data	No data (increase in demented vs. control, overlap between groups)	No.<br>(SDS effects possible)
Upadhaya et al. 2012[18]	Hypertonic .32M sucrose, 1M TBS lysate.<br>IP 6E10, B10AP or A11 with LDS elution	>1000 kDa on blue native gel (smaller on SDS PAGE)	EM spheroids & protofibrils with 6E10 immunogold after LDS elution	No data	No.<br>(detergent effects possible)<br>(APP binding possible)
Um et al. 2012[19]	TBS lysate, 175k x g sup., preclear, then PrP IP	No data.	No data<br>(binds to PrP 23-111)	Activates Fyn in mouse cortical cultured neurons	No, but unlikely as minimally manipulated.
Esparza et al.	PBS lysate, 17k &	>670 kDa on	No data	No data	Yes: spiked

2013[1]	100k x g sup.	SEC at pH7.4		(ratio of oligomer to plaque distinguishes demented from nondemented subjects with plaques: no overlap between groups)	monomer controls resulted in no aggregation. HJ3.4 antibody confirmed to not detect APP.
Lesne et al. 2013[20]	1) TBS + 0.01% NP40, 0.1% SDS, 0.1% Triton lysate 2) TBS lysate	12, 27, 56, 110 kDa on SDS PAGE WB in detergent extracts. No Aβ*56 in TBS extracts	No data (Binds 6E10 & A11)	No data (Aβ*56 higher in control than AD, but overlap between groups)	No (detergent effects possible) (incomplete antibody specificity)
Um et al. 2013[21]	TBS lysate, 100k x g sup., preclear protein A, preclear Fc.	No data	No data	Induces calcium oscillations in cultured neurons. Not in PrP ko or mGluR5 ko neurons	No, but unlikely as minimally manipulated.
Dohler et al. 2014[22]	TBS+0.5% NP40 +0.5% deoxycholate lysates, 100k x g sup., Superdex 200 SEC in PBS+1% Sarcosyl	wide range 17-300 kDa in SEC followed by 6E10 WB	No data (Co-IP with anti-PrP)	No data	No. (Detergent effects possible) (APP binding possible)
Mc Donald et al. 2015[23]	TBS lysate, 150 k x g sup., SEC, then protein A preclear, IP & elution in 2%SDS.	Several: 158-670, 44-158, ~7 kDa on SEC superdex 75, or 75-200 kDa in ammonium acetate & Asym flow field flow fractionation.	No data, (falls apart into 7 kDa species with reduced N-terminal antigenicity in 6M Guan, 885 mM FA, 6M Urea)	No data	Partial. Ammonium Acetat vs. saline: High MW oligomers in both. (SDS effects possible)
Esparza et al (This communication)	PBS+0.45% CHAPS lysate, 17k then 100k x g sup, then 475 k x g pellet, then IP with ammonium hydroxide elution	>670 kDa by SEC in TBS pH 7.4	Clusters of 10-20 nm ovoids by immunoEM using HJ3.4	No data	Yes. Spiked monomer controls resulted in no aggregation. IP & elution does not change size forms. HJ3.4 antibody does not detect APP.

Abbreviations:

6E10: monoclonal antibody that binds both Aβ and APP

A11: polyclonal antibody that binds oligomeric aggregates of many proteins, no specific for Aβ

AFM: atomic force microscopy

APP: amyloid precursor protein

CNBr: cyanogen bromide

EM: electron microscopy

g: force of gravity, as a unit for relative centrifugal force

hMSC: human mesenchymal stem cells

icv: intracerebroventricular

IP: immunoprecipitation

k: kilo (1000)

kDa: kilodaltons

ko: knockout

LDS: lithium dodecyl sulfate detergent

LTD: long term depression

LTP: long term potentiation

MALDI: Matrix assisted laser desorption/ionization mass spectrometry

PAGE: polyacrylamide gel electropheresis

PBS: phosphate buffered saline

pI: isoelectric point

PrP: prion protein

SDS: sodium dodecyl sulfate detergent

SEC: size exclusion chromatography

Sup: supernatant

TBS: tris buffered saline

TEM: transmission electron microscopy

WB: Western blot

SUPPLEMENTARY DISCUSSION

As noted in the main manuscript, there are multiple differences between our method for isolating and purifying soluble Aβ aggregates from human brain compared with previous approaches.

1) We used an ELISA-based method to quantify soluble Aβ aggregates that does not cause *ex vivo* aggregation. In contrast, methods involving SDS such as gel electrophoresis likely cause both *ex vivo* aggregation and disaggregation, making them inaccurate reflections of the quantity and size of the aggregates.
2) We quantitatively tracked the loss of soluble Aβ aggregates at each step in the procedure, and used albumin blocking to prevent nonspecific loss. Previous preparative approaches that did not involve quantitative bookkeeping or systematic albumin blocking likely resulted in substantial and unknown amounts of nonspecific loss of soluble Aβ aggregates. Thus, the properties of the soluble Aβ aggregates previously characterized may have reflected those of minority species that were especially resistant to nonspecific binding.
3) We have detected *bona fide* full length Aβ in the soluble aggregates, based on mass spectrometry. In contrast, previous methods used antibodies with incomplete specificity (e.g. 6E10 which binds to amyloid precursor protein fragments, A11 which binds to oligomeric forms of other proteins). Previous mass spectrometric characterization was limited to mid-domain and C-terminal digested peptides, which could also have arisen from amyloid precursor protein fragments.

There are many unanswered questions arising from the data presented:

Are there other classes of soluble Aβ aggregates without canonical N-termini exposed? We can envision in theory several fundamentally distinct types of soluble Aβ aggregates: A) Aggregates with two or more canonical Aβ N-termini exposed, which would have been detected by the methods used here; B) Aggregates with only one canonical Aβ N-terminal exposed, which would not have been quantified by our ELISA but still could have been immunoprecipitated and detected by electron microscopy. Indeed, this could explain why some of the soluble Aβ aggregates in our preparation had only 1 immunogold label (**Fig. 6E**); C) Aggregates with no canonical Aβ N-termini exposed, which could have been immunoprecipitated if the HJ5.1 epitope (mid domain) were exposed but would not have been detected by any of the methods used here. Thus, a priority for future research will be a broader exploration of the spectrum of Aβ aggregates present in the human AD brain using other detection reagents. We are in the process of characterizing antibodies to other Aβ epitopes (including truncated and post-translationally modified forms), and we plan to repeat the immunoprecipitations and immunoelectron microscopic characterizations using additional appropriate antibodies. A recent report indicates that the peri-plaque Aβ species in transgenic mouse brains appear to be quite heterogeneous with regard to antibody binding, with only partially overlapping subsets binding several aggregate-selective and epitope specific antibodies [24].

Why does the low molecular weight fraction contain a few particles which bind 2 anti-Aβ antibodies on electron microscopy, while this fraction yielded no detectible signal on the HJ3.4-HJ3.4 soluble Aβ aggregate ELISA? This could represent an incomplete sensitivity of the ELISA, or alternatively could indicate that these species have both Aβ epitopes locked in a relatively rigid conformation with both facing the same direction. A (hypothetical) species with 2 epitopes held in a relatively rigid parallel orientation during ELISA could be bound to the capture antibody but not by the detection antibody, accounting for a potential false negative result on our ELISA.

Does the CHAPS used in our extraction procedure alter structural or functional properties of the soluble Aβ aggregates? Additional preparations using larger amounts of tissue but excluding CHAPS will be required to address this concern.

Why are the electron microscopic-based sizes of the soluble Aβ aggregates seen in our preparation larger than those seen in Noguchi et al [9] ? It is possible that sub-aggregates were broken apart Noguchi et al's preparation or clustered together during ours.

Why are portions of the surfaces of the soluble Aβ aggregates not immunoreactive with the anti-Aβ antibody? It is possible that there are other protein constituents, or Aβ species that do not have intact N-termini and therefore would not be immunoreactive with HJ3.4. Further investigations will be needed using a panel of monoclonal antibodies directed at different Aβ epitopes as well as other protein constituents once they are identified.

What is the relationship between soluble Aβ aggregates and tau pathology? Increasing evidence implicates tau pathology as a close correlate of neurodegeneration in Alzheimer's disease [25-27] http://www.alzforum.org/news/conference-coverage/tau-pet-studies-agree-tangles-follow-amyloid-precede-atrophy. Tau pathology may be downstream of Aβ [28,29] in transgenic mice, where tau is required for APP transgene-related behavioral deficits [30] likely via tau coupling to the kinase Fyn [31]. However, investigations of the mechanisms underlying the relationship between Aβ aggregates and tau pathology have mainly relied on synthetic Aβ aggregate preparations and transgenic mice. The effects of human AD brain-derived soluble Aβ aggregates on tau pathology remain to be determined and represents an important future direction.

SUPPLEMENTARY REFERENCES

1 Esparza, T. J. *et al.* Amyloid-beta oligomerization in Alzheimer dementia versus high-pathology controls. *Annals of neurology* **73**, 104-119, doi:10.1002/ana.23748 (2013).

2 Roher, A. E., Ball, M. J., Bhave, S. V. & Wakade, A. R. Beta-amyloid from Alzheimer disease brains inhibits sprouting and survival of sympathetic neurons. *Biochemical and biophysical research communications* **174**, 572-579 (1991).

3 Frackowiak, J., Zoltowska, A. & Wisniewski, H. M. Non-fibrillar beta-amyloid protein is associated with smooth muscle cells of vessel walls in Alzheimer disease. *Journal of neuropathology and experimental neurology* **53**, 637-645 (1994).

4 Kuo, Y. M. *et al.* Water-soluble Abeta (N-40, N-42) oligomers in normal and Alzheimer disease brains. *The Journal of biological chemistry* **271**, 4077-4081. (1996).

5 Roher, A. E. *et al.* Morphology and toxicity of Abeta-(1-42) dimer derived from neuritic and vascular amyloid deposits of Alzheimer's disease. *The Journal of biological chemistry* **271**, 20631-20635 (1996).

6 McLean, C. A. *et al.* Soluble pool of Abeta amyloid as a determinant of severity of neurodegeneration in Alzheimer's disease. *Annals of neurology* **46**, 860-866 (1999).

7 Gong, Y. *et al.* Alzheimer's disease-affected brain: presence of oligomeric A beta ligands (ADDLs) suggests a molecular basis for reversible memory loss. *Proceedings of the National Academy of Sciences of the United States of America* **100**, 10417-10422, doi:10.1073/pnas.1834302100 (2003).

8 Shankar, G. M. *et al.* Amyloid-beta protein dimers isolated directly from Alzheimer's brains impair synaptic plasticity and memory. *Nature medicine* **14**, 837-842 (2008).

9 Noguchi, A. *et al.* Isolation and characterization of patient-derived, toxic, high mass amyloid beta-protein (Abeta) assembly from Alzheimer disease brains. *The Journal of biological chemistry* **284**, 32895-32905, doi:10.1074/jbc.M109.000208 (2009).

10 Mc Donald, J. M. *et al.* The presence of sodium dodecyl sulphate-stable Abeta dimers is strongly associated with Alzheimer-type dementia. *Brain : a journal of neurology* **133**, 1328-1341, doi:10.1093/brain/awq065 (2010).

11 Barry, A. E. *et al.* Alzheimer's disease brain-derived amyloid-beta-mediated inhibition of LTP in vivo is prevented by immunotargeting cellular prion protein. *The Journal of neuroscience : the official journal of the Society for Neuroscience* **31**, 7259-7263, doi:10.1523/JNEUROSCI.6500-10.2011 (2011).

12 Freir, D. B. *et al.* Interaction between prion protein and toxic amyloid beta assemblies can be therapeutically targeted at multiple sites. *Nature communications* **2**, 336, doi:10.1038/ncomms1341 (2011).

13 Lasagna-Reeves, C. A., Glabe, C. G. & Kayed, R. Amyloid-beta annular protofibrils evade fibrillar fate in Alzheimer disease brain. *The Journal of biological chemistry* **286**, 22122-22130, doi:10.1074/jbc.M111.236257 (2011).

14 Zou, W. Q. *et al.* Amyloid-beta42 interacts mainly with insoluble prion protein in the Alzheimer brain. *The Journal of biological chemistry* **286**, 15095-15105, doi:10.1074/jbc.M110.199356 (2011).

15 Jin, M. *et al.* Soluble amyloid beta-protein dimers isolated from Alzheimer cortex directly induce Tau hyperphosphorylation and neuritic degeneration. *Proceedings of the National Academy of Sciences of the United States of America* **108**, 5819-5824, doi:10.1073/pnas.1017033108 (2011).

16 Bjorklund, N. L. *et al.* Absence of amyloid beta oligomers at the postsynapse and regulated synaptic Zn2+ in cognitively intact aged individuals with Alzheimer's disease neuropathology. *Molecular neurodegeneration* **7**, 23, doi:10.1186/1750-1326-7-23 (2012).

17 McDonald, J. M., Cairns, N. J., Taylor-Reinwald, L., Holtzman, D. & Walsh, D. M. The levels of water-soluble and triton-soluble Abeta are increased in Alzheimer's disease brain. *Brain research* **1450**, 138-147, doi:10.1016/j.brainres.2012.02.041 (2012).

18 Upadhaya, A. R., Lungrin, I., Yamaguchi, H., Fandrich, M. & Thal, D. R. High-molecular weight Abeta oligomers and protofibrils are the predominant Abeta species in the native soluble protein fraction of the AD brain. *Journal of cellular and molecular medicine* **16**, 287-295, doi:10.1111/j.1582-4934.2011.01306.x (2012).

19 Um, J. W. *et al.* Alzheimer amyloid-beta oligomer bound to postsynaptic prion protein activates Fyn to impair neurons. *Nature neuroscience* **15**, 1227-1235, doi:10.1038/nn.3178 (2012).

20 Lesne, S. E. *et al.* Brain amyloid-beta oligomers in ageing and Alzheimer's disease. *Brain : a journal of neurology* **136**, 1383-1398, doi:10.1093/brain/awt062 (2013).
21 Um, J. W. *et al.* Metabotropic glutamate receptor 5 is a coreceptor for Alzheimer abeta oligomer bound to cellular prion protein. *Neuron* **79**, 887-902, doi:10.1016/j.neuron.2013.06.036 (2013).
22 Dohler, F. *et al.* High molecular mass assemblies of amyloid-beta oligomers bind prion protein in patients with Alzheimer's disease. *Brain : a journal of neurology* **137**, 873-886, doi:10.1093/brain/awt375 (2014).
23 Mc Donald, J. M. *et al.* The aqueous phase of Alzheimer's disease brain contains assemblies built from approximately 4 and approximately 7 kDa Abeta species. *Alzheimer's & dementia : the journal of the Alzheimer's Association* **11**, 1286-1305, doi:10.1016/j.jalz.2015.01.005 (2015).
24 Pickett, E. K. *et al.* Non-Fibrillar Oligomeric Amyloid-beta within Synapses. *Journal of Alzheimer's disease : JAD* **53**, 787-800, doi:10.3233/JAD-160007 (2016).
25 Scholl, M. *et al.* PET Imaging of Tau Deposition in the Aging Human Brain. *Neuron* **89**, 971-982, doi:10.1016/j.neuron.2016.01.028 (2016).
26 Johnson, K. A. *et al.* Tau positron emission tomographic imaging in aging and early Alzheimer disease. *Annals of neurology* **79**, 110-119, doi:10.1002/ana.24546 (2016).
27 Ossenkoppele, R. *et al.* Tau PET patterns mirror clinical and neuroanatomical variability in Alzheimer's disease. *Brain : a journal of neurology* **139**, 1551-1567, doi:10.1093/brain/aww027 (2016).
28 Gotz, J., Chen, F., van Dorpe, J. & Nitsch, R. M. Formation of neurofibrillary tangles in P301l tau transgenic mice induced by Abeta 42 fibrils. *Science* **293**, 1491-1495 (2001).
29 Oddo, S., Billings, L., Kesslak, J. P., Cribbs, D. H. & LaFerla, F. M. Abeta immunotherapy leads to clearance of early, but not late, hyperphosphorylated tau aggregates via the proteasome. *Neuron* **43**, 321-332 (2004).
30 Roberson, E. D. *et al.* Reducing endogenous tau ameliorates amyloid beta-induced deficits in an Alzheimer's disease mouse model. *Science* **316**, 750-754 (2007).
31 Ittner, L. M. *et al.* Dendritic function of tau mediates amyloid-beta toxicity in Alzheimer's disease mouse models. *Cell* **142**, 387-397 (2010).
